# Synthesis and biological evaluation of ibuprofen/o-vanillin Schiff base complexes with anti-inflammatory, anti-proliferative and anti-SARS-COV-19 activities

**DOI:** 10.1038/s41598-026-38270-8

**Published:** 2026-03-07

**Authors:** Laila H. Abdel-Rahman, Doaa Abou El-ezz, Abdel-Mawgoud M. Abdel-Mawgoud, Mohamed R. Shehata, Mahmoud Abd El Aleem Ali Ali El-Remaily, Mohamed Abdel-Hameed, Shaaban K. Mohamed

**Affiliations:** 1 Chemistry Department, Faculty of Science, Sohag University, Sohag, 82534 Egypt; 2 Pharmacology and Toxicology Department, Faculty of Pharmacy, October University for Modern Sciences & Arts, Giza, Egypt; 3 Chemistry Department, Faculty of Science, Cairo University, Giza, Egypt; 4 Chemistry and Environmental Division, Manchester Metropolitan University, Manchester, M1 6GD England; 5 Faculty of Science, Sohag National University, Sohag, Egypt

**Keywords:** Ibuprofen, o-vanillin, Antimicrobial, Anticancer, DNA interaction, DFT, Vanadium, Copper, Nickel, Zinc, Biochemistry, Chemical biology, Chemistry, Computational biology and bioinformatics, Drug discovery

## Abstract

This study presents the synthesis and comprehensive characterization of four novel metal-complexes formed from the reaction of copper(II), nickel(II), zinc(II), and vanadium(IV) salts with the bidentate ibuprofen Schiff base ligand (HL) in 1:1 metal-to-ligand ratio. The copper complex shows the highest antibacterial and cytotoxicity efficiency, in addition to a strong binding interactions with DNA. The *in-vitro* anti-inflammatory and anti-COVID-19 potencies of HL and its complexes were assessed, ranking them as: CuL > NiL > ZnL > VOL > HL.

**Supplementary Information:**

The online version contains supplementary material available at 10.1038/s41598-026-38270-8.

## Introduction

The formation of metal complexes designed for various applications is a well-explored field of study. First reported by Hugo Schiff in 1864, Schiff bases are azomethine-based multifunctional ligands that exhibit strong coordination with a wide range of metals^1^. These compounds are synthesized through the condensation of an active carbonyl molecule, such as an aldehyde or ketone, with a primary amine, typically under azeotropic distillation conditions^2^. Recently, there has been a surge of interest in Schiff bases due to their extensive potential applications in chemical synthesis, catalysis, magnetism, and chemosensors^3^. This increased interest stems from the ability of Schiff bases to vary in denticity, shape, and size, as well as the presence of various substituents that can either donate or withdraw electrons. Their biological activities are notable, with Schiff bases demonstrating antifungal, antibacterial, antimalarial, antiproliferative, and anti-inflammatory effects^4^.

Nonsteroidal anti-inflammatory drugs (NSAIDs) are commonly used to alleviate pain and inflammation resulting from illness or injury. Increasing evidence suggests a correlation between NSAID use and a decreased risk of developing various cancers, with in vitro studies indicating significant anticancer potential. This compounds may inhibit the production of prostaglandins through mechanisms other than cyclooxygenase (COX) in cultured cancer cells, thereby influencing cell proliferation and apoptosis^5^.

Ibuprofen, a widely utilized nonsteroidal anti-inflammatory drug, is effective in treating fever and pain. However, oral administration of ibuprofen carries certain risks, including gastrointestinal irritation and bleeding. The electron density of the Schiff base ligand is influenced by methoxy groups (-OCH₃), which act as electron-donating substituents. This increased electron density can enhance the ligand’s binding affinity for metal ions, resulting in stronger metal–ligand coordination complexes. Methylated Schiff bases often exhibit improved biological activity, as the presence of methyl groups can enhance their lipophilicity, facilitating interactions with biological targets such as enzymes and receptors. Furthermore, methoxy groups can alter the electronic absorption characteristics, fluorescence, and NMR spectra of Schiff bases. Consequently, methoxy Schiff bases may prove useful as catalysts or sensors where specific spectroscopic properties are desired.

The significance of metal-Schiff base complexes is largely dependent on the type of metal ions involved. Copper-Schiff base complexes are known to undergo redox reactions and are frequently employed as catalysts in oxidation and reduction processes. These copper complexes are particularly recognized for their antibacterial and anticancer properties due to their ability to generate reactive oxygen species (ROS) and interact with biomolecules. Nickel-Schiff bases can serve as catalysts in both industrial and biological processes by mimicking the active sites of certain metalloenzymes. Owing to their electrochemical stability, nickel complexes are often utilized in electrochemical reactions, such as fuel cells and water splitting. Zinc, a multifunctional trace element, plays a crucial role in numerous physiological functions, including metabolism, immune response, cognitive function, cell growth and development, and reproduction. Notably, zinc has shown positive effects in the prevention and treatment of various diseases, including cancer. Over the past decade, a variety of vanadium compounds have emerged as anti-inflammatory therapeutic metallodrugs targeting different diseases. Recent studies indicate that specific vanadium species are involved in immune-driven molecular processes that regulate and influence immune responses^4^.

Given the importance of developing anti-inflammatory drugs with minimal side effects, the exploration of ibuprofen Schiff bases as potential agents for detecting and identifying small amounts of various metal ions is particularly relevant.

In this study, we aim to investigate the pharmacological properties of four new ibuprofen Schiff base complexes: CuL, NiL, ZnL, and VOL. We will assess their cytotoxic, anti-inflammatory, and antibacterial efficacies, as well as their affinity for CT-DNA. Additionally, molecular modeling studies will be conducted to elucidate the potential binding processes of these drugs with COVID-19 and cyclooxygenases, thereby corroborating the biological findings from our experimental investigations. In light of these considerations, we present a novel synthetic route for the ibuprofen Schiff base ligand, characterized as a water-insoluble Schiff base, and its corresponding complexes. Furthermore, we will discuss their stoichiometric ratios, thermal stability, cytotoxicity, anti-inflammatory activity, DNA binding capabilities, PXRD, molecular docking analysis against the target proteins PDB: 6lu7 (SARS-CoV-2) and PDB: 6COX, along with density functional theory calculations.

## Experimental

In this research, components from Merck, Fluke, and Sigma-Aldrich were utilized without any additional purification. Notice: in this article we abbreviated the Schiff base ligand as HL for simplicity.

### Instrumentation and materials

In our study, we exclusively utilized high-purity analytical-grade substances for all reagents, including solvents and metal salts. The metal salts employed were zinc nitrate hexahydrate [Zn(NO₃)₂·6H₂O], vanadyl acetylacetone [VO(acac)₂], copper chloride dihydrate [CuCl₂·2H₂O], and nickel nitrate hexahydrate [Ni(NO₃)₂·6H₂O]. We obtained CT-DNA and Tris(hydroxymethyl)aminomethane from in-house resources, while 2-Hydroxy-3-methoxybenzaldehyde was sourced from Sigma-Aldrich. Ibuprofen was provided by the Egyptian government’s Central Drug Authority.

To determine the melting points of these compounds, we employed a Gallenkamp instrument (UK). For microanalytical data concerning carbon, hydrogen, and nitrogen, we relied on a Perkin-Elmer 240c elemental analyzer. Ultraviolet–visible spectra were obtained using a Shimadzu UV mini-1240 spectrophotometer. The Fourier transform infrared spectroscopy (FT-IR) analysis of the synthesized ligand and its corresponding metal complexes was performed using an FT-IR Alpha Bruker instrument, with KBr pellets at a resolution of 4 cm^−1^. Nuclear magnetic resonance (NMR) spectra of the ligand were recorded in deuterated dimethyl sulfoxide (DMSO-d₆) utilizing a 400 MHz Bruker BioSpin spectrometer.

To measure the molar conductivity of the metal complexes at a concentration of 1 × 10^–3^ M in N,N-dimethylformamide (DMF), we used the Jenway model 4510 conductivity meter. Mass spectra were obtained with Thermo Scientific’s GCMS ISQ model, which features a direct inlet system. X-ray powder diffraction (XRD) analyses were conducted on a Bruker D8 Advance diffractometer using copper K-alpha radiation (λ = 1.5406 Å).

For assessing the pH-dependent absorbances of 1 × 10^−3^ M CuL, NiL, ZnL, and VOL complexes, we utilized Britton-Robinson buffers to maintain precise pH values. pH readings were obtained using a Jenway 3510 pH meter. All thermogravimetric analyses (TGA) were carried out at Azhar University’s Microanalysis Center under a nitrogen atmosphere, using a Shimadzu TGA-60H analyzer. The gas flow rate was set at 40 mL/min, with samples heated at 50°C/min from ambient temperature up to 1000°C. This center was also responsible for evaluating the antibiotic efficacy of our synthesized compounds ^6–8^.

### Synthesis of ibuprofen ethyl ester and ibuprofen hydrazide

In a round-bottomed flask, a mixture of Ibuprofen (0.01 mol, 2.06 g) and absolute ethanol (20 mL) with the addition of the catalyst sulphuric acid (0.5 mL) was refluxed for 12 h and neutralized with 10% sodium bicarbonate to pH 8. Then the TLC was run for the reaction mixture using a mixture eluents ( ethyl acetate : n-hexane (3:7)) showed R_f_ value = 0.65.  Yield: (91%) pale yellow oil; B. p. 263–265 °C, FT-IR spectrum, ʋ cm^-1^: 1735 (CO),1165 (C-O ester)^9^. In a 100 mL round-bottomed flask, ibuprofen ethyl ester (0.02 mol, 4.69g) was added, followed by hydrazine hydrate 99% (0.1 mL) and 30 mL of absolute ethanol. The reaction mixture was then refluxed for 30 hours, and concentrated to abought a fifth of its original bulk. The ibuprofen hydrazide was then preciptated as white crystals. Yield: 89%, m.p.74 °C. R_f_ value (ethyl acetate : n-hexane 3:7) = 0.83; FT-IR (KBr) spectrum, ʋ cm^−1^: 1685 (CO amide),3313, 3278.9 (asym. & sym.NH_2_).

### Synthesis of ibuprofen Schiff base

**Fig. 1:**
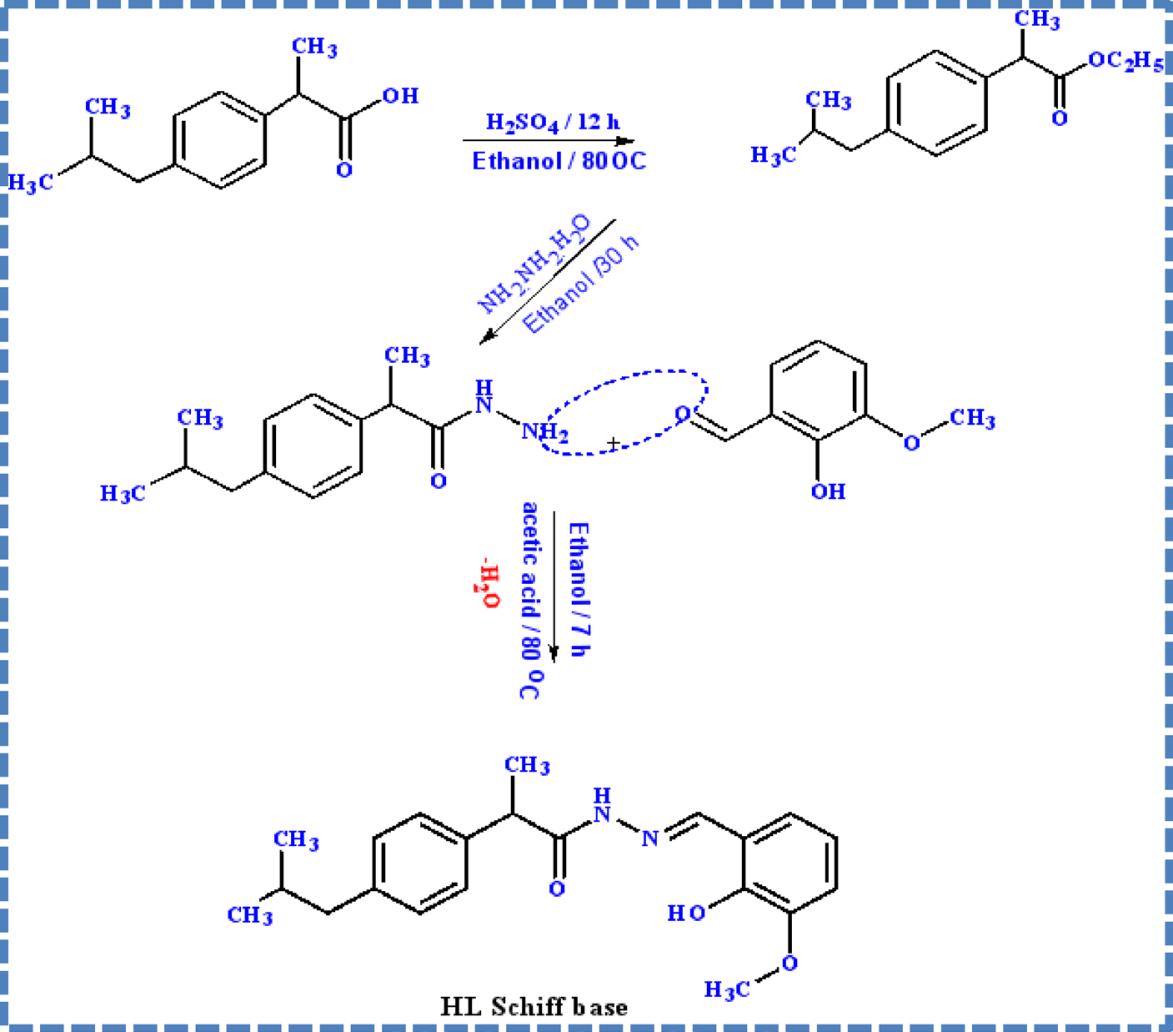
Synthesis of HL [Ibuprofen hydrazide with o-vanillin (2-Hydroxy-3-methoxy benzaldehyde)]. Notice: HL is the Schiff base ligand abbreviation we use in this article for simplicity,

The synthesis of the HL Schiff base ligand involves combining 1.101 g of ibuprofen hydrazide, equating to 5 mmol, in 15 ml of chloroform with 0.76 g of o-vanillin, also 5 mmol, dissolved in 15 ml of methanol. This mixture is then subjected to reflux for a duration of one hour at 60 degrees Celsius. The reaction is facilitated by the addition of a catalytic amount of acetic acid. A pale-yellow precipitate forms upon completion of the reaction, which is then collected by filtration. The precipitate is washed and dried over anhydrous calcium chloride under vacuum conditions. The ligand’s empirical formula is determined to be C_21_H_26_N_2_O_3_, with a melting point of 171 degrees Celsius. For X-ray crystallographic analysis, single crystals are obtained by recrystallization using an equimolar solution of methanol and chloroform. The molecular weight of this compound is 354.33 g per mole. This process is outlined in Fig. 1.

### Synthesis of Schiff base metal complexes

**Fig. 2:**
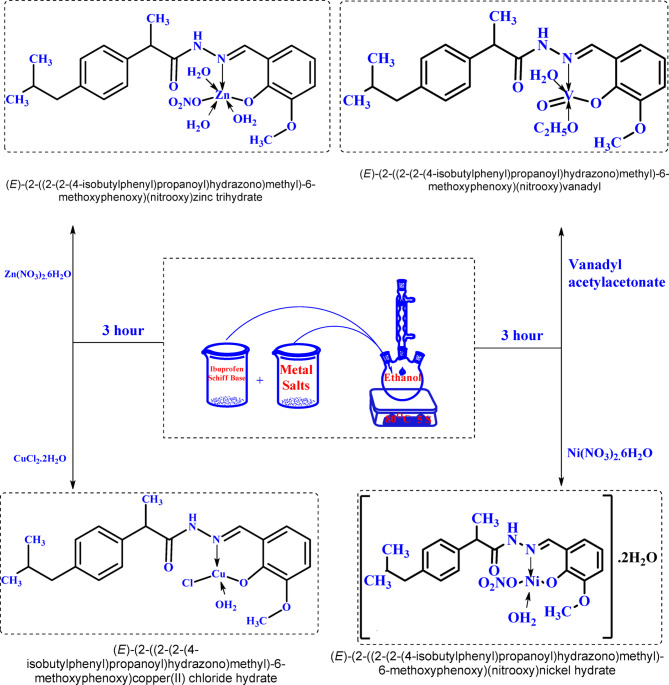
Synthesis of CuL, NiL, ZnL, and VOL ibuprofen Schiff base complexes.

In separate flasks, each containing 20 mL of ethanol, 1 mmol (0.35 g) of the HL Schiff base was reacted with 1 mmol of specific metal salts individualy. The metal salts used included zinc nitrate hexahydrate [Zn(NO₃)₂·6H₂O, 0.297 g], vanadyl acetylacetonate [VO(acac)₂, 0.265 g], copper chloride dihydrate [CuCl₂·2H₂O, 0.170 g], and nickel nitrate hexahydrate [Ni(NO₃)₂·6H₂O, 0.291 g], as illustrated in Fig. 2.

These mixtures were subjected to vigorous agitation through magnetic stirring for 3 h at a temperature of 60 °C. On completion, the resulting solid complexes were filtered off, washed throughly and dried under vaccum as pure product. All products were then dried in a desiccator with anhydrous calcium chloride. The complexes were thoroughly analyzed and evaluated their properties and characteristics.

### Spectral data for HL ligand and its metal complexes

**HL ligand;** Yield 86%; *m.p*.171⁰C.; Pure yellow. Anal. Calc. for C_21_H_26_N_2_O_3_ (%): C, 71.1; H, 7.34; N, 7.89. Found [%]: C, 71.23; H, 7.58; N, 7.85. M. Wt. Calc (g/mol) 354.4, Found 354.33. FT-IR [KBr, cm^−1^]: 1612 υ_St_(-CH = N), 1248 υ_Ph_(-C-O), 1704.

υ_Ph_(-C = O), 3280 υ_Ph_(-N–H). ^1^H-NMR [400MHz, CDCl_3_] ppm δ = 0.83–0.86 (d, 6H), 1.37–1.43 (d,3H), 1.45–1.84 ( m -1H), 2.37–2.52 ( d,2H), 3.65–3.70 ( q–1H ), 3.80–3.82 (s, 3H), 6.81–7.30 (M–7 H), 8.41(s,1H), 10.82(s,1H), 11.73(s,1H). ^13^C-NMR (75MHz, CDCl_3_) δ = 18.88, 19.10, 22.64, 30.10, 39.55, 39.97, 40.38, 44.70, 56.28, 113.19, 114.12, 118.34, 119.68, 121.12, 129.46, 139.72, 140.14.

**ZnL complex;** Yield 82%, dark purple solid. Anal. Calc. for **C**_**21**_**H**_**31**_**N**_**3**_**O**_**9**_**Zn** (%): C, 47.11; H, 5.79; N, 7.85. Found (%): C, **47.23**; H, **6.15**; N, **8.27**. M. Wt. calc (g/mol) **534.89**, Found 534.36. FT-IR (KBr, cm^−1^): 971 υ (OH)/H_2_O, 1608υ_St_ (-CH = N), 1245υ_Ph_ (-C-O), 550υ(M–O), 457υ(M–N).

**CuL complex;** Yield 79%; Reddish brown solid. Anal. Calc. for **C**_**21**_**H**_**28**_**N**_**2**_**O**_**4**_**CuCl** (%): C, 49.85; H, 6.11; N, 5.51, Cl, 7.54. Found (%): C, 49.62; H, 6.34; N, 5.79; Cl, 7.57 .M. Wt. Calc (g/mol) 508.34, Found 505.86. FT-IR [KBr, cm^−1^]: 970υ (OH)/H_2_O, 1604υ_St_ (-CH = N), 1247υ_Ph_ (-C-O), 520υ(M–O), 442υ(M–N).

**NiL complex;** Yield 80%; Dark green solid. Anal. Calc. for **C**_**21**_**H**_**31**_**N**_**3**_**O**_**9**_**Ni** (%): C, 47.73; H, 5.87; N, 7.95. Found (%): C, 47.92; H, 6.20; N, 8.23. M.wt.Calc (g/mol) 528, Found 528.06. FT-IR [KBr, cm^−1^]: 967 υ (OH)/H_2_O, 1600υ_St_ (-CH = N), 1240υ_Ph_ (-C-O), 560υ(M–O), 436υ(M–N).

**VOL complex;** Yield 79%; Black solid. Anal. Calc. for **C**_**23**_**H**_**32**_**N**_**2**_**O**_**6**_**V** (%): C, 57.09; H, 6.62; N, 5.79. Found [%]: C, 57.29; H, 6.74; N, 6.03. M.wt. Calc (g/mol) 483.45, Found 484.13. FT-IR (KBr, cm^−1^): 973 υ (OH)/H_2_O, 1602υ_St_ (-CH = N), 1252υ_Ph_ (-C-O), 527υ(M–O), 422υ(M–N).

### Complexes in solution state

An investigation was conducted to examine the stability and molar weight of NiL, CuL, ZnL, and VOL complexes when dissolved in a solvent. The research employed Job’s techniques, including both continuous variation and molar ratio methods, to explore these properties of the metal ions in their complex forms^10^.

After achieving equilibrium in the reaction combinations (M&L), the absorbance of all mixtures was measured. The data obtained from these measurements enabled a comparison of the absorbance values relative to the mole fractions of the ligand, expressed as mole {[L]/([L] + [M])}, or the molar proportions of ligand to metal, represented as [L]/[M]. This analysis provided valuable insights into the interaction dynamics between the substances involved in the reaction^11^.

### Thermogravimetric analysis (TGA) and Thermo-kinetic parameters

The thermal stability of the newly formed compounds and the coordination sphere of the solvent were evaluated using Thermogravimetric Analysis (TGA). This methodology was conducted under a nitrogen atmosphere over a temperature range of 25 to 1000 °C, with the aim of determining the metal content within these innovative molecular structures. Additionally, the process is kinetically regulated to reveal both the kinetic and thermodynamic properties of the complexes. The supplementary data includes the mathematical formulations associated with the Coats-Redfern method^1,2^.

### DFT studies

The Gaussian 09 software was utilized to investigate the most stable conformations of the innovative molecules, employing the B3LYP/GENECP theoretical framework. This approach incorporated carbon (C), hydrogen (H), nitrogen (N), oxygen (O), chlorine (Cl), and bromine (Br) atoms, which were described using the 6-311G +  + [d, p] basis set. In contrast, the metal atoms were treated with the LANL2DZ basis set to ensure accurate geometric optimization^3–5^.

### Biological studies

#### DNA binding experiments

All assays were performed using a Tris–HCl buffer solution with a concentration of 55 mM and a pH of 7.4. Prior to the experiments, CT-DNA was purified via centrifugal dialysis to ensure its integrity. The methods employed for the interaction studies of the complexes with DNA were consistent with those outlined in earlier publications^6,7^.

#### Anticancer activity

Spectrophotometric analysis was performed at 564 nm using an “ELIZA” microplate reader (Meter tech. 960) to determine the absorbance, or optical density, of each sample. The study investigated the cytotoxic effects of an ibuprofen Schiff base ligand and its corresponding metal complexes on various cancer cell lines, including colon cancer cells (HCT-116), breast cancer cells (MCF-7), and hepatic cancer cells (HepG-2). The procedures employed for this investigation closely followed those described in previous studies^8–10,12,13^.

#### Antimicrobial performance of the ligand and its metal complexes

##### Antibacterial activity.

The well diffusion method was used to assess the antibacterial and antifungal properties of HL Schiff base ligand and its CuL, NiL, ZnL, and VOL complexes against a range of bacterial strains, including *Staphylococcus aureus* and *Bacillus subtilis*, which are classified as Gram-positive, as well as *Escherichia coli* and *Proteus vulgaris* and the fungal strains Candida albicans and Aspergillus fumigatus. Dimethyl sulfoxide (DMSO) was used as a comparative baseline at concentrations of 15 mg/mL and 25 mg/mL to ensure it did not exhibit any antibiotic effects, as it was solely employed as a solvent to dissolve the compounds under investigation. According to the study, this negative control did not inhibit bacterial growth at the specified concentrations. Gentamycin and Ketoconazole were the standard antibacterial and antifungal testing drugs. Mueller Hinton agar was used as the growth medium for each strain of microorganism. Every chemical was made at 1000 ppm concentrations in DMSO solution. To guarantee accuracy, each test was carried out three times, and the average value was calculated using a microbiological scale, the inhibitory zone was measured in millimeters (mm). Each serially diluted solution was supplemented with the resuscitated bacterial and fungal strains, which were then incubated for 24 h at 37 °C. The inhibitory zones formed around the test samples were measured in millimeters and compared with those of the positive control, Gentamycin, which served as a reference standard in the experiment. This approach was guided by established procedures described in the literature^1,11,14,15^.

##### Antifungal activity

Using the disc diffusion method, the ligand and its novel complexes were evaluated for their efficacy against fungal infections caused by two distinct species: Candida albicans and Aspergillus fumigatus. According to existing literature, the purification of the fungal cultures was achieved through single-spore isolation. The sensitivity of these fungi to the antifungal agents was assessed by measuring the diameters of the inhibitory zones during the experimental process process^8,16,17^.

#### Anti-inflammatory activity

Using an egg albumin assay, we evaluated the in vitro anti-inflammatory capabilities of ibuprofen and its CuL, NiL, ZnL, and VOL complexes formed with a Schiff base ligand. In each test tube, we added 2 mL of dimethyl sulfoxide (DMSO) at four varying concentrations: 100, 200, 300, and 400 μM. This was combined with 2.8 mL of phosphate buffer (pH 6.4) and 0.2 mL of egg albumin. The mixtures underwent denaturation by heating in a water bath at 70 °C for 20 min, following a 30-min incubation period at 37°C. After cooling, we measured the absorbance at 660 nm.

Using Eq. 1, we deduced that typical ibuprofen exhibited a comparable level of protein denaturation inhibition. The percentage of inhibition is calculated using the formula:

1 $${\text{inhibition\% }} = \left( {\left( {{\mathrm{A}}_{0} - {\mathrm{A}}_{{\mathrm{m}}} } \right)/{\mathrm{A}}_{0} } \right) \times 100$$

where A₀ represents the absorbance of the control sample and Aₘ denotes the absorbance of the test sample. The IC₅₀ value was computed for all samples; this value indicates the specific concentration at which 50% of the maximum protein denaturation inhibition occurs^18^.

### Molecular docking

Employing the Gaussian 09 [G09] tool, the molecular geometry of the specified ligand as well as its complexes with copper, nickel, zinc, and vanadium (denoted as CuL, NiL, ZnL, and VOL, respectively) were meticulously modeled and saved in the widely-used PDB format. Additionally, the human DNA receptor with the PDB identifier 6lu7 [SARS-CoV-2] and the crystal structure of the main protease of the same virus, 6COX, were retrieved from the Protein Data Bank for further analysis^19^.

## Results and discussion

A new ligand was synthesized through the reaction of ibuprofen hydrazide and o-vanillin in an ethanolic medium, resulting in a distinctive Schiff base compound. Subsequent reactions of this HL ligand with various metal salts, including copper, nickel, zinc, and vanadium, led to the formation of the complexes CuL, NiL, ZnL, and VOL. These metal-Schiff base derivatives demonstrate remarkable non-hygroscopic characteristics and exceptional thermal stability.

### Molar conductivity and elemental analysis

Examining Table 1, we observe an array of physical attributes, microanalytical findings, and molar conductivity values related to the ligand under investigation and its resulting complexes. The data indicates that each of these complexes exhibits a consistent 1:1 stoichiometry, maintaining a constant metal-to-ligand ratio throughout^20^.

**Table 1:** Physicochemical properties of the **HL** ligand and its metal complexes; color, melting point (m. p °C/ dec. temp), M. wt., and magnetic moment (μ eff, B.M).

Compound formula	Color	M.P °C/ Dec. Temp	μ eff [B.M.]	M. Wt	Analysis: found [calc.]		
					C[%]	H[%]	N[%]
CuL C_21_H_28_N_2_O_4_CuCl	Reddish brown	429	2.08	470.34	47.73 (47.92)	5.87 (6.20)	7.95 (8.23)
NiL C_21_H_31_N_3_O_9_Ni	Dark green	268	Dia	528.00	47.73 (47.92)	5.87 (6.20)	7.95 (8.23)
ZnL C_21_H_31_N_3_O_9_Zn	Dark purple	360	Dia	534.89	47.11 (47.23)	5.79 (6.15)	7.85 (8.27)
VOL C_23_H_33_N_2_O_6_V	Black	215	1.94	483.45	57.09 (57.29)	6.62 (6.74)	5.79 (6.03)
HL C_21_H_26_N_2_O_3_	Yellow	171	-	354.44	71.10 (71.23)	7.34 (7.58)	7.89 (7.85)

Complexes displaying non-electrolytic properties were identified with molar conductivity values of 1 × 10^−3^ M. These substances exhibited a negligible tendency to conduct electricity in aqueous solutions, suggesting that they do not significantly dissociate into ions. This characteristic can be attributed to their molecular structure and bonding, which typically involves nonpolar covalent bonds. The low conductivity observed in this range is indicative of molecular compounds that remain intact when dissolved in water, as opposed to electrolytes, which dissociate and enable the movement of charged particles that facilitate electrical current flow.

Non-electrolytic substances are of particular interest in various scientific fields, including chemistry, biology, and environmental studies, due to their unique interactions with solutions and the absence of ionic contributions to solution conductance.

### Magnetic measurement

The distinct geometric forms of the VOL and CuL complexes, characterized by a square pyramidal configuration and a tetrahedral structure, are mirrored in their respective effective magnetic moments (μeff) of 1.94 and 2.08 B.M. For ZnL, its diamagnetic nature, which arises from a filled 3d10 electronic configuration, suggests a tetrahedral or octahedral configuration. To clarify the precise three-dimensional architecture of the ZnL complex, various spectroscopic techniques may be employed. The NiL complex, on the other hand, exhibits a square planar geometry, a characteristic that is consistent with its diamagnetic properties. These findings are further substantiated by spectroscopic and analytical investigations.

### ^1^H NMR spectra of HL and its ZnL and NiL complexes

The ^1^H NMR spectroscopic analysis of the ligand [HL] and its diamagnetic zinc complex [ZnL] was conducted in the deuterated solvent DMSO-d6, employing tetramethylsilane [TMS] as the internal reference standard. The ligand’s spectrum presented a distinct singlet at δ 8.60 ppm, which is ascribed to the azomethine proton [–CH=N–]. Various aromatic protons manifested as signals in the range of δ 6.82 to δ 7.30 ppm. Additionally, a sharp singlet at δ 11.73 ppm and δ 10.82 ppm represented the ligand’s phenolic proton and its adjacent [–NH–] group, respectively^21^. For the zinc complex [ZnL], the 1H NMR spectrum displayed a notable singlet at δ 8.32 ppm, which corresponds to the azomethine proton (-CH = N-)^22–24^. A series of signals between δ 6.74 and δ 7.13 ppm was attributed to the aromatic protons; these shifts in peak positions suggest the formation of the ZnL complex. It’s worth noting that the ligand’s phenolic proton signal at δ 10.82 ppm was absent in the ZnL complex’s spectrum. This is likely due to the participation of the phenolic oxygen atom in the coordination environment of the zinc center. The spectra of these compounds can be found in the supplementary material as Figure S1.

### FT-IR spectral studies

The Fourier Transform Infrared (FT-IR) spectroscopy method was employed to identify the specific functional groups of the ligand and to provide insights into the nature of interactions between these groups and the central metal ions present in the newly formed complexes. A comprehensive overview of the characteristic FT-IR spectral bands for both the HL ligand and its derived complexes—CuL, NiL, ZnL, and VOL—can be found in Figure S2 and Table S1.

The FT-IR spectrum of the HL ligand displayed a notable peak at 1612 cm^−1^, indicative of the v(CH = N) stretching vibration. Upon coordination with metal ions, this peak shifted to lower wavenumbers, ranging from 1608 to 1600 cm^−1^ in the spectra of the CuL, NiL, ZnL, and VOL complexes. This observation suggests the successful formation of the respective chelates and an alteration in the ligand’s electronic environment due to metal ion binding.

The presence of intermolecular hydrogen bond interactions [-NH-O-] in the HL ligand can be inferred from the two weak absorption peaks observed at 3040 and 3192 cm^−1^. These bands imply a potential bonding arrangement between the phenolic [-OH] and the ibuprofen hydrazide nitrogen [-C = N] groups, leading to the formation of either enol or keto forms of the isomers. Additionally, a shift in the v(C-O) vibration from 1248 cm^−1^ in the isolated ligand to a range of 1240–1252 cm^−1^ in the metal complexes was detected. This shift indicates that the ligand interacts with the metal ions through a monodentate coordination mode.

The identification of the counteranions within the complexes was facilitated by several key considerations, as established by Nakamoto. For instance, a monodentate nitrate group (NO₃^−^), which has C₂V symmetry, typically exhibits three distinct nitrogen–oxygen stretching vibrations. In the examined samples, the ZnL complex displayed these bands at 1446 cm^−1^, 1293 cm^−1^, and 1007 cm^−1^, while the NiL complex exhibited similar but slightly shifted bands at 1438 cm^−1^, 1312 cm^−1^, and 1032 cm^−1^. These spectral variations indicate the unique bonding interactions occurring between the ligand and the respective metal ions.

Furthermore, the spectrum of the VOL complex indicated v(V = O) vibrations at 992 cm^−1^, while the CuL complex spectra revealed chloride v(Cu-Cl) vibrations at 350 cm^−1^. Lastly, the emerging peaks in the range of 520–560 cm^−1^ and 422–457 cm^−1^ were attributed to v(M–O) and v(M–N) vibrations, respectively. The presence of new peaks between 1600 and 1612 cm^−1^ is indicative of the successful coordination of the HL ibuprofen Schiff base ligand to CuL, NiL, ZnL, and VOL metal ions, with bonding occurring through the azomethine nitrogen and phenolic oxygen atoms.

### Electronic spectra

The outcomes of various structural examination techniques are often assessed through the analysis of molecular electronic absorption spectra. These spectra provide valuable data that aid in determining the properties and characteristics of substances by measuring the absorption of light at different wavelengths. Evaluating these spectra is crucial for understanding molecular composition and structure, which is essential in numerous scientific and industrial fields. The spatial configuration of metal ions within complexes is discerned through the analysis of their electronic spectra, which involves observing the specific locations and intensities of d-d transition bands. This technique enables the determination of the metal’s stereochemical arrangement within the molecular structure.

At a temperature of 298 Kelvin and across wavelengths ranging from 200 to 800 nm, the electronic absorption spectra for the HL ligand and its resulting complexes are depicted in Supplementary Figure S3 and summarized in Table 2. The ligand exhibits absorption bands in the ultraviolet to visible spectrum at 288 nm, indicative of π-π* electronic transitions, and at 355 nm, associated with the ligand’s imine moiety, denoted as n → π*.

**Table 2:** UV- Vis. spectra; wavelength (λ _max_, nm) of the HL ligand and its complexes.

Compounds	λ_max_ (nm)λ	Abs	ε_max,_ (dm^3^ Mol^-1^ cm)	Assignment
HL	288	1.855	1855	$\uppi \to {\uppi }^{*}$
	355	1.927	1927	$\mathrm{n}\to {\uppi }^{*}$
VOL	290	1.826	1826	$\uppi \to {\uppi }^{*}$
	360	2.355	2355	$\mathrm{n}\to {\uppi }^{*}$
	436	2.379	2379	MLCT
	552	0.335	335	^2^B_1g_ (d_z_^2^) → ^2^E and ^2^T_2g_ → ^2^E_g_
NiL	290	1.841	1841	$\uppi \to {\uppi }^{*}$
	359	2.384	2384	$\mathrm{n}\to {\uppi }^{*}$
	448	2.585	2585	MLCT
	746	0.5	500	LMCT band ^1^A_1g_ → ^1^A_2g_ and ^1^A_1g_ → ^1^E_g_
CuL	289	1.848	1848	$\uppi \to {\uppi }^{*}$
	360	2.376	2376	$\mathrm{n}\to {\uppi }^{*}$
	448	2.526	2526	^2^B_1g_ → ^2^B_2g_ and
	504	0.584	584	^2^B_1g_ → ^2^E_g_
ZnL	289	1.847	1847	$\uppi \to {\uppi }^{*}$
	359	2.376	2376	$\mathrm{n}\to {\uppi }^{*}$
	442	2.566	2566	MLCT
	510	0.569	569	LMCT band

Upon complexation with metal ions, these bands shift to higher energy levels, as presented in Table 2. The emergence of absorption bands centered at 436–448 nm in the metal complexes suggests a transfer of charge from the ligand to the metal ion, a phenomenon known as metal-to-ligand charge transfer (MLCT). Furthermore, the new NiL complex displays a significantly low-energy absorption band at 746 nm, characteristic of the ligand-to-metal charge transfer (LMCT) process, contributing to a broad and extensive absorption profile for the complex.

### Mass spectra

Mass spectrometric analysis of the HL ligand and its corresponding metal complexes with copper (Cu), nickel (Ni), zinc (Zn), and vanadium (V) has been conducted to elucidate the coordination framework of the synthesized ligand. The mass spectrum of the HL ligand displays a distinct molecular ion peak at m/z 354.44, which aligns with the molecular formula C₂₁H₂₆N₂O₃. Additional peaks at m/z 354.33, 161.20, and 105.26 indicate fragmentation patterns and provide insights into the ligand’s stability.

For the CuL complex, mass spectrometry reveals molecular ion peaks at m/z 505.5, consistent with the composition C₂₁H₃₁N₂O₆CuCl and a theoretical molecular weight of 508.34 amu. This confirms the 1:1 metal-to-ligand ratio. Moreover, the presence of a peak at m/z 370.48, corresponding to the ligand’s weight at m/z 354.33, supports the structural integrity of the complex.

Similarly, the mass spectrum of the NiL complex shows molecular ion peaks at m/z 528.06, which aligns with the formula C₂₁H₃₁N₃O₉Ni and a predicted molecular weight of 528.00 amu. A peak at m/z 353.68, equivalent to the ligand’s molecular weight, confirms the complex’s 1:1 stoichiometry.

**Fig. 3:**
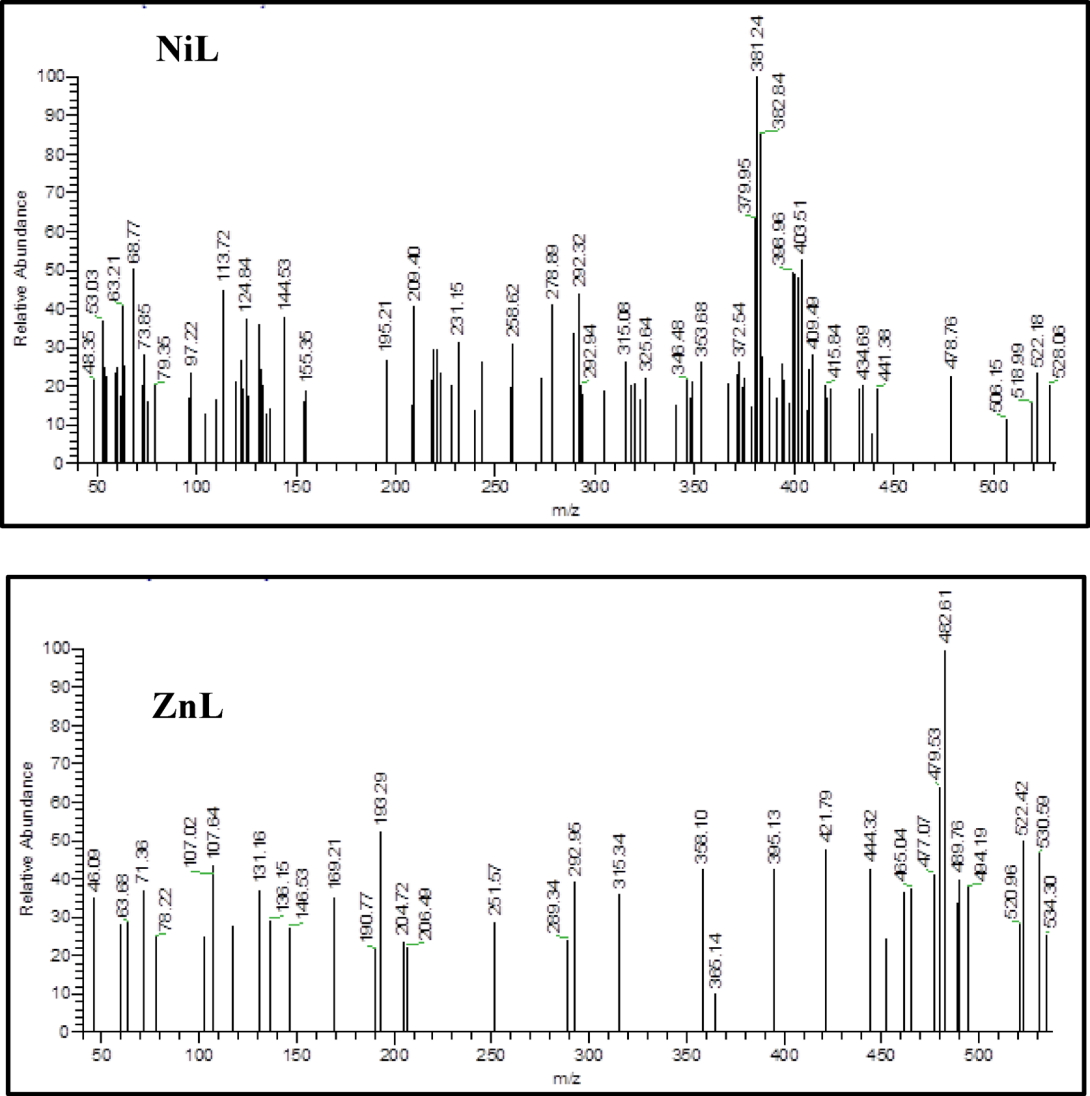
The mass spectra of NiL and ZnL complexes.

In the case of the ZnL complex, the mass spectrum reveals molecular ion peaks at m/z 534.36, corresponding to the formula C₂₁H₃₁N₃O₉Zn, and aligns with the anticipated molecular weight of 534.89 amu, thus validating the 1:1 metal-to-ligand ratio. The presence of a peak at m/z 358.10, similar to the ligand’s weight at m/z 354.33, reinforces the structure of the complex (Fig. 3).

**Fig. 4:**
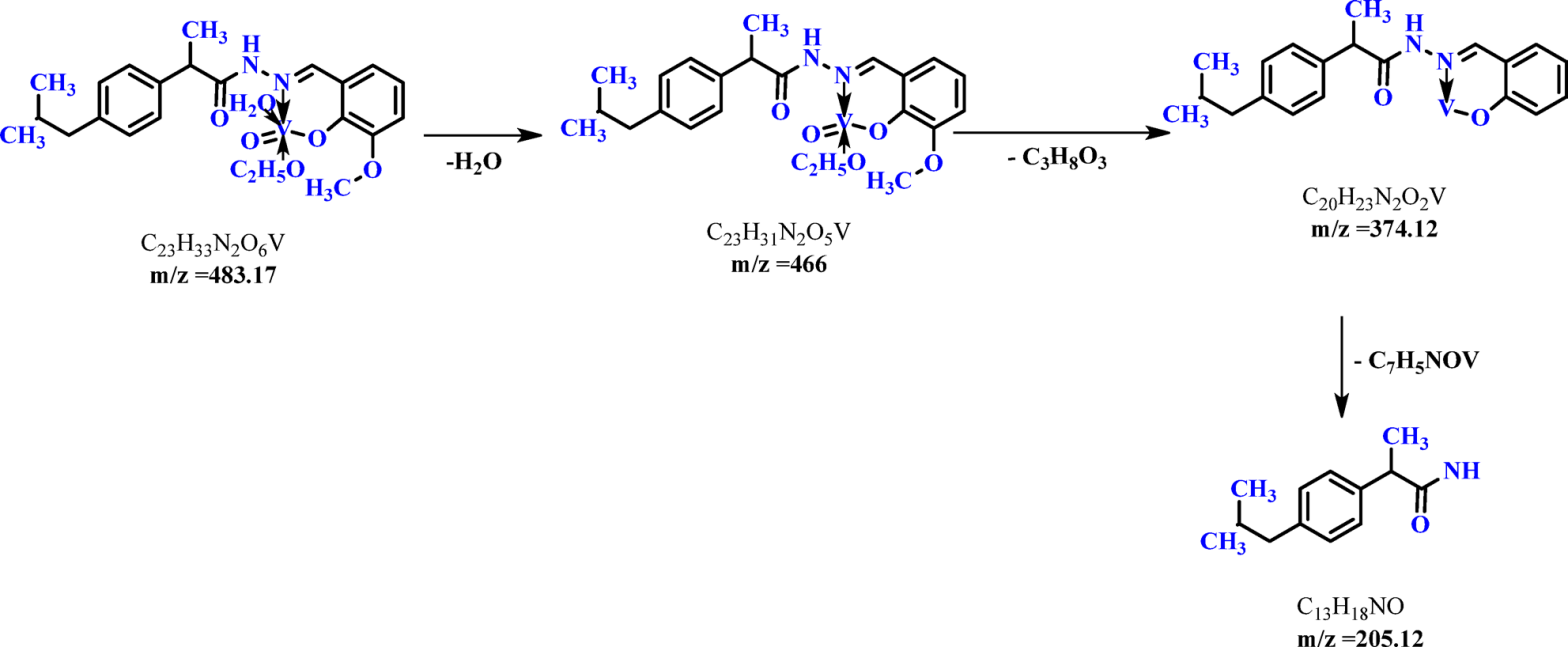
The degradtion of VOL ibuprofen Schiff base complex.

Finally, the mass spectrum of the VOL complex displays molecular ion peaks at m/z 484.13, corresponding to the formula C₂₃H₃₂N₂O₆V, which is consistent with the calculated molecular weight of 483.45 amu, confirming the 1:1 metal-to-ligand stoichiometry. A peak at m/z 360.68, linked to the ligand’s weight at m/z 354.33, further substantiates the structure of the complex. The mechanism is illustrated in Fig. 4.

The observed mass spectral data harmonize with the expected carbon, hydrogen, and nitrogen values, corroborating the proposed formulas. Supplementary information can be found in Fig. 3 and Figure S4^25^.

### Dynamic stability of prepared complexes

**Fig. 5:**
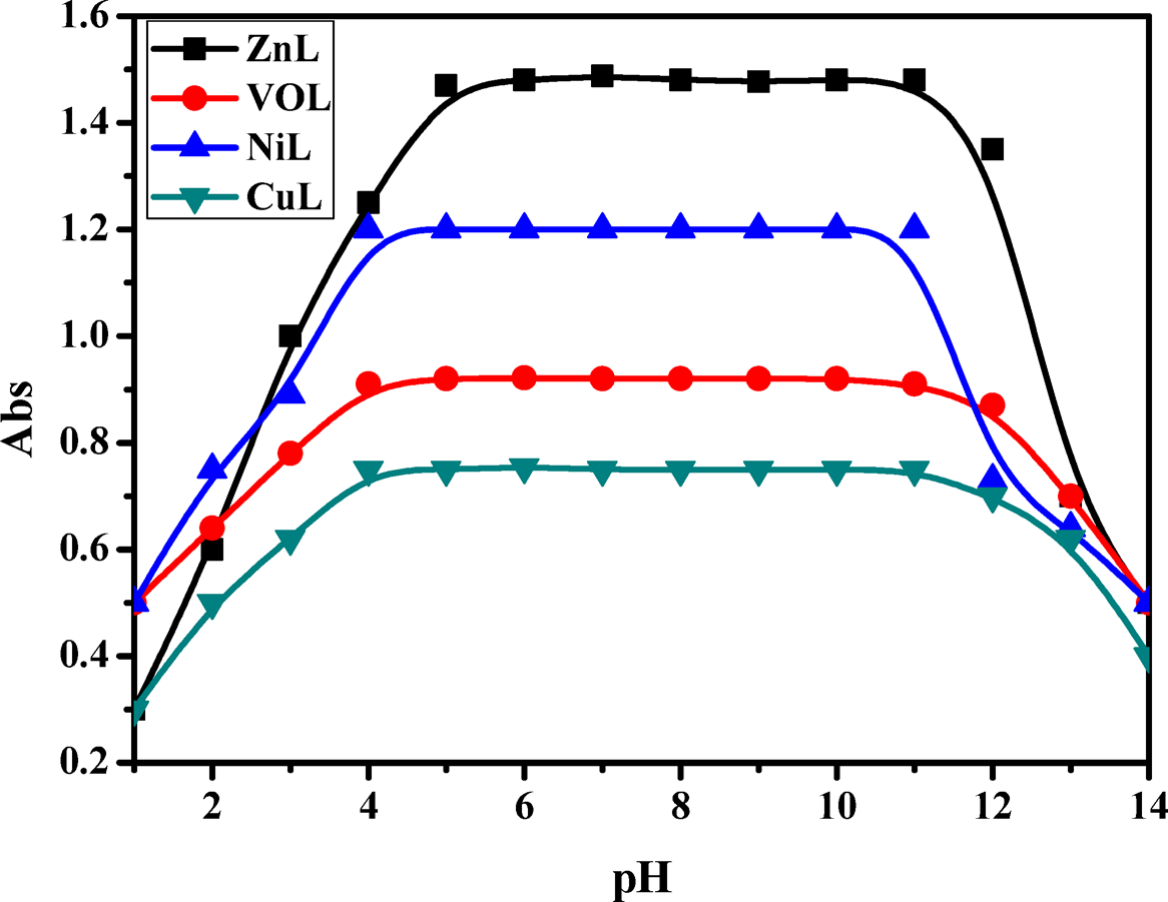
The curve of the profile of the synthesized HL complexes at [complex] = 10^-3^M in DMF at 298K.

The visual representation in Fig. 5 clearly demonstrates the remarkable stability of the newly developed substances throughout an extensive pH spectrum. This characteristic implies that when utilizing these compounds to form metal complex chelates, it is advisable to operate within the pH span of 4 to 12 to ensure optimal performance and effectiveness^10,26^.

### Complexes stoichiometry

Employing Job’s spectroscopic approach, we systematically examined the complex formation process, as depicted in Figure S5. The spectroscopic data revealed that the complexes exhibited peak absorbance within the range of 504 to 710 nm, which is indicative of an optimal mole fraction for the ligand, denoted as X ligand, at approximately 0.55. This outcome suggests a balanced interaction, where the metal ion forms a 1:1 [M:L] complex with the **HL** ligand. Additionally, the observed wavenumbers of the complexes spanned from 14,085 to 19,841 cm^−1^, further corroborating the established stoichiometry and the nature of the metal–ligand bonding within the molecular structure.

### Thermogravimetric analysis [TGA] and Thermo-kinetic parameters

Thermogravimetric analysis (TGA) was employed to assess the thermodynamic stability of the newly synthesized compounds and to gather data on the presence of solvent molecules within the crystalline framework of these complexes. The results from both thermogravimetric (TG) and differential thermogravimetric (DTG) studies, conducted across a temperature range of 25 to 1000 degrees Celsius under a nitrogen atmosphere, are compiled in Table 3 and visualized in Figure S6 for all the new metal-based formations.

**Table 3:** Thermal decomposition steps, mass loss (%), proposed lost segments, final residue thermo-kinetic activation parameters of each decomposition step for the prepared complexes in an N_2_ atmosphere, at a heating rate of 5 °C* / min*.

Complexes	Temperature ^o^C	Fragment loss %		Weight loss %		E* [KJmol^-1^]	A [S^-1^]	∆H* [Jmol^-1^]	∆G* [KJmol^-1^]	∆S* [Jmol^-1^K^-1^]
		Lost form	M. Wt	Found	Calc					
CuL	44–429 °C	H_2_O coord + CH_3_ + Cl	68.5	13.47	13.42	8.2	17.2	4223.8	23,300.9	-4163
	430–803 °C	C_19_H_14_N_2_O_3_	318	62.78	62.91			5250.26	26,527.3	-51.75
	804–999 °C	**1/2C** + 4H_2_	14	2.75	2.77			7404.67	40,349.5	-52.1
	$>999 ^\circ \mathrm{C}$	Cu + **1/2C**	69.5	13.93	13.75			7416.67	41,349.5	-53.1
NiL	46–268 °C	2H_2_O hyd + H_2_O coord. + NO_3_ + 3H_2_	122	23.19	23.11	2.3	3.1	3621	19,421	-53.94
	269–593 °C	C_19_H_8_O_2_N_2_	296	56.05	56.06			4832	25,231	-50.196
	594–995 °C	C_2_H_11_O	51	9.42	9.66			8721	46,021	-51.8675
	$>995 ^\circ \mathrm{C}$	Ni	58.69	11.34	11.12			1100	5782	-55.25
ZnL	44–360 °C	3H2O coord + NO3 + C6H12O	216	40.31	40.38	6.2	8.3	4301	24,002	-55.93
								6203	5531	-54.18
	360–718 °C	C_11_N_2_O_2_	192	36.16	35.9			2438	12,321	-54
	719–999 °C	C_4_H_13_	61	11.91	11.4			6236	44,521	-54
	$>999$ °C	Zn	65.4	11.63	12.2			6660	45,521	-54.21
VOL	45–215 °C	H_2_O coord + C_2_H_5_ + 3H_2_	53	11	10.96	8.2	17.2	4423.8	23,326.9	-5163
	215–515 °C	C_9_H_7_N_2_O	159	32.84	32.89			5250.26	26,527.3	-51.75
	516–708 °C	C_8_H_5_O_2_	133	27.85	27.5			7404.67	40,349.5	-52.1
	709–978 °C	C_4_H_7_O	71	15.38	14.8			7416.67	41,349.5	-53.1
	$>978 ^\circ \mathrm{C}$	VO	66.9	12.93	13.8			3621	19,421	-53.94

The TGA of the CuL complex [CuL(H₂O)(Cl)] reveals three distinct stages of weight reduction. The initial stage, occurring between 44 and 429 degrees Celsius, corresponds to a loss of 13.47% of the sample’s weight (theoretically predicted: 13.42%), indicating the removal of one coordinated water molecule, CH₃, and Cl components. The second stage, at temperatures from 430 to 803 degrees Celsius, shows a weight loss of 62.78% (calculated: 62.91%), consistent with the decomposition of the C₁₉H₁₄N₂O₃ fragment. The final process, ranging from 804 to 999 degrees Celsius, results in a weight loss of 2.75% (calculated: 2.77%), attributed to the elimination of the C₂H₁₁O fragment, ultimately leaving copper(I) chloride as the residue.

For the NiL complex [NiL(H₂O)(NO₃)]·2H₂O, the TGA curve reveals three weight-loss events. The first, occurring from 46 to 268 degrees Celsius, shows a weight loss of 23.19% (calculated: 23.11%), consistent with the release of two molecules of H₂O, the coordinated water, and the nitrate group. The second event, from 269 to 593 degrees Celsius, corresponds to a weight loss of 56.05% (calculated: 56.06%), which matches the decomposition of the C₁₉H₈O₂N₂ segment. The last stage, between 594 and 995 degrees Celsius, demonstrates a weight loss of 9.42% (calculated: 9.66%), consistent with the loss of the C₂H₁₁O fragment, leaving nickel metal as the residue.

Similarly, the ZnL complex [ZnL(H₂O)₃(NO₃)] undergoes three weight-loss phases. The first phase, between 44 and 360 degrees Celsius, involves a weight reduction of 40.31% (calculated: 40.38%), which corresponds to the elimination of three water molecules, nitrate, and C₆H₁₂O groups. The second phase, from 360 to 718 degrees Celsius, shows a weight loss of 36.16% (calculated: 35.9%), corresponding to the degradation of the C₁₁N₂O₂ fragment. The final phase, from 719 to 999 degrees Celsius, results in a weight loss of 11.91% (calculated: 11.4%), associated with the removal of the C₄H₁₃ fragment, leaving zinc metal as the residue.

The TGA of the VOL complex [VOL(H₂O)(C₂H₅O)] presents four weight-loss steps. The first step, occurring between 45 and 215 degrees Celsius, leads to a weight loss of 11.00% (calculated: 10.96%), attributed to the removal of the coordinated water, C₂H₅, and three H₂ parts. The second step, from 215 to 515 degrees Celsius, results in a weight loss of 32.89% (calculated: 32.80%), consistent with the breakdown of the C₉H₇N₂O fragment. The third step, from 516 to 708 degrees Celsius, shows a weight decrease of 27.85% (calculated: 27.5%), corresponding to the loss of the C₈H₅O₂ group. The last step, from 709 to 978 degrees Celsius, shows a weight loss of 15.38% (calculated: 14.8%), coinciding with the decomposition of the C₄H₇O fragment, yielding VO as the final residue.

When applying the Coats-Redfern equation to determine the thermodynamic activation parameters for these decomposition processes, the observed data suggest that all processes are endothermic, as indicated by positive activation energy values^27,28^. These new structures exhibit a negative activation entropy, suggesting a higher degree of organization compared to the reactants, which implies a delay in the onset of the reaction. The initial stages of the reaction may be sluggish due to bond polarization and electronic transitions occurring in the active phase.

The degradation reactions of the metal complexes are characterized by significantly positive values of the free energy change (ΔG*), indicating that the degradation steps are not spontaneous, with the free energy of the original molecule being higher than that of the final degradation product. The low A values associated with these complexes result in an extended pyrolysis process. As shown in Figure S6, the magnitude of E* suggests that these reactions involve various rotational, translational, and vibrational states, along with variations in mechanical potential energy^20,29,30^.

### PXRD study

**Fig. 6:**
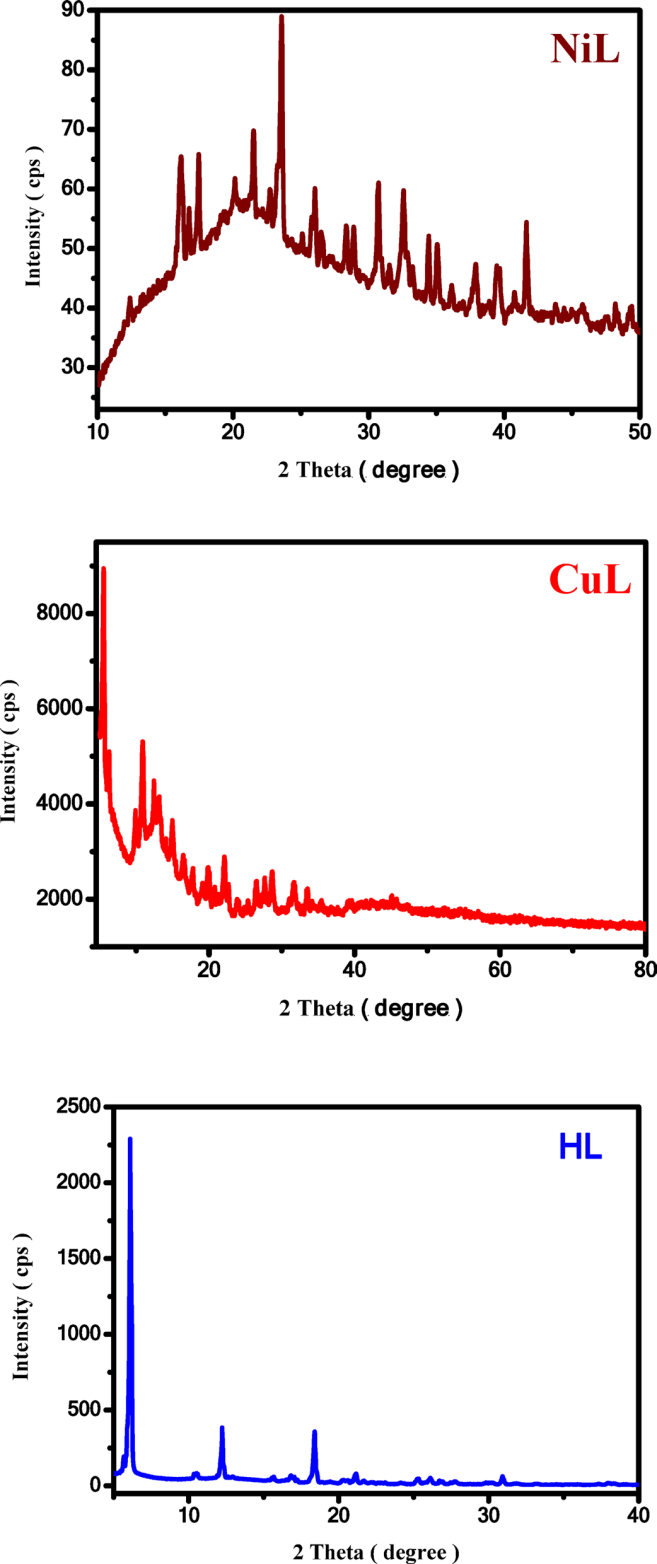
PXRD patterns of the NiL and CuL complexes and HL ligand.

The crystalline nature of the Schiff base ligand and its derived complexes was assessed through an analysis of their respective powder X-ray diffraction (PXRD) patterns. It was observed that, with the exception of the ZnL and VOL complexes, the CuL and NiL complexes, along with the HL ligand, exhibited distinct crystalline structures. These complexes and the ligand were found to have unit cell dimensions characterized by unequal values for the lengths of the 'a', 'b', and 'c' axes, as well as distinct angles between them, denoted as 'α', 'β', and 'γ'. These details are presented in Fig. 6, Eq. 2, and in the Supplementary Information (Figure S7). The typical crystallite size for these substances was estimated using the Debye–Scherrer formula based on the XRD pattern data^31^.

2 $$\upxi = \frac{{K\uplambda }}{{_{{\upbeta 1/2}} \cos\uptheta }}$$

For the specified angle [θ], considering X-ray radiation with a wavelength of [1.5425 Å], the peak width was determined using a K-constant of 0.95, which is typically applied for organic substances. The parameter β₁/₂ represents the half-maximum width of the reference diffraction peak in radians. This information was essential for estimating the dislocation density within the crystalline structure.

To determine the average particle diameter (ξ), the parameter δ, which represents the full width at half maximum, was calculated using a specific formula. This method is standard for analyzing crystalline materials and provides insights into the particle size distribution and overall crystalline quality, as shown in Eq. 3:

3 $${\updelta } = \frac{1}{{\upxi ^{2} }}$$

For the Schiff base ligand and its Ni(II) and Cu(II) complexes, the value of δ ranges from 5.52 to 49.21 nm. The unit cell parameters of the NiL complex are as follows: (a = 9.20000, \text{Å}), (b = 8.65800, \text{Å}), (c = 12.65400, \text{Å}), with a space group of P 1 21/c 1 (14), indicating a monoclinic crystal lattice.

In contrast, the unit cell parameters of the CuL complex are (a = 16.42000, \text{Å}), (b = 8.54500, \text{Å}), (c = 19.00100, \text{Å}), also with a space group of P 1 21/c 1, and a monoclinic crystal lattice.

The obtained unit cell parameters for the HL ligand are (a = 21.45620, \text{Å}), (b = 21.45680, \text{Å}), and (c = 4.30950, \text{Å}), with a space group of P 1 and a triclinic crystal lattice^21,32^.

### X-ray structure analysis

#### Crystal structures of HL ligand

**Fig. 7:**
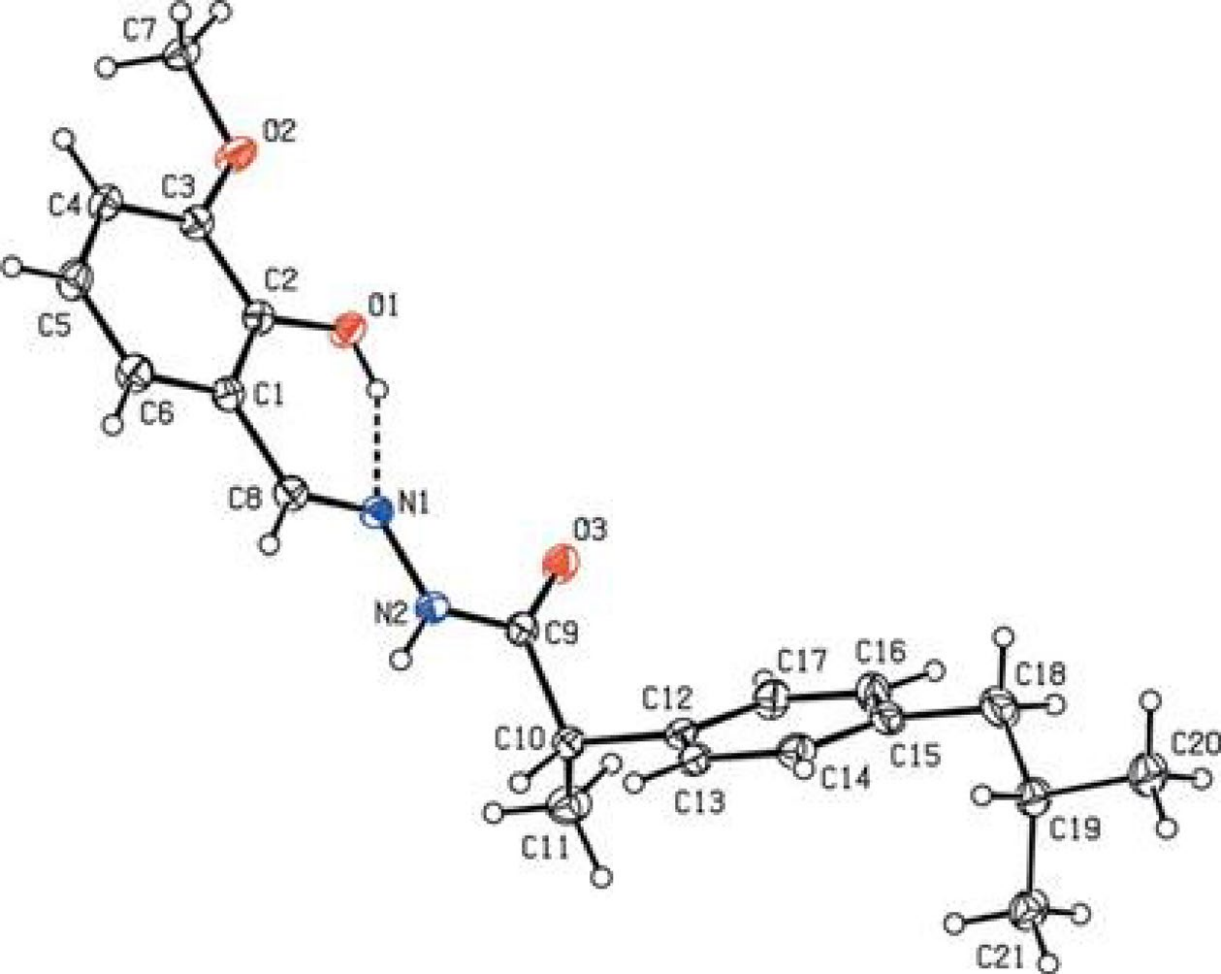
The title molecule with labeling figure and 30% probability level ellipsoids. The intramolecular O—H N hydrogen bond is indicated as a dashed line. Only the major component of the disorder is shown.

In the crystal, N2—H2 O2 and weaker N2—H2 O1 hydrogen bonds (Table 1) form chains of molecules extending along the *c*-axis direction Fig. 7. The molecular packing was provided by normal van der Waals interactions between chains.

#### Supramolecular features and Hirschfeld surface analysis

**Fig. 8:**
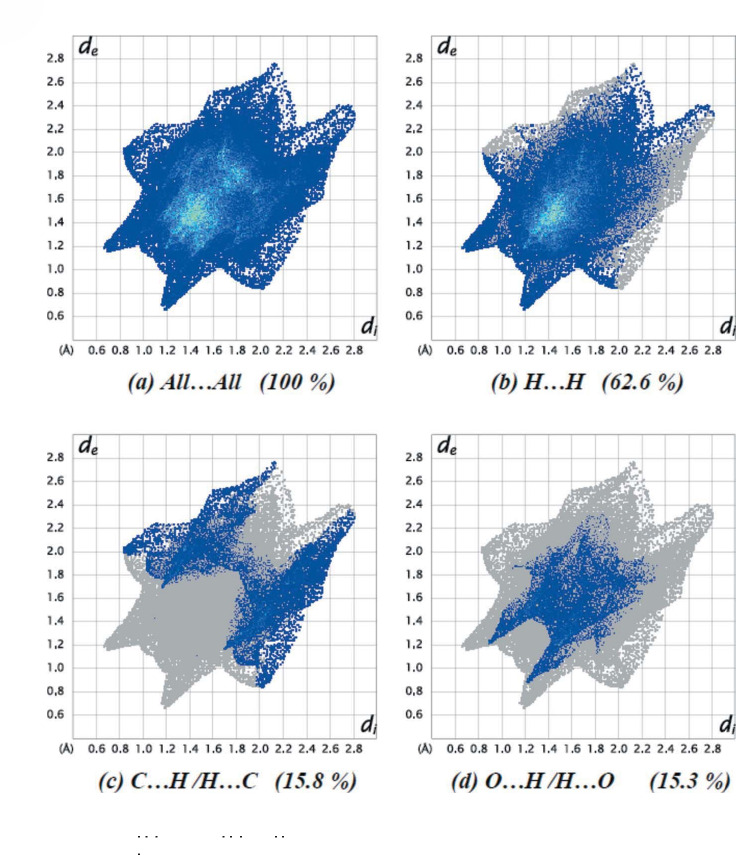
Two-dimensional ﬁngerprint plots for the title compound, showing (*a*) all interactions, delineated into (*b*) H H, (*c*) C H/H C, and (*d*) O H/H O interactions. The *d*_i_ and *d*_e_ values are the closest internal and external distances (in A) from given points on the Hirschfeld surface.

Hirschfeld surfaces and their related two-dimensional fingerprint plots were generated using *CrystalExplorer17.5* to visually represent the intermolecular interactions in the crystal structure of the title compound. The Hirschfeld surface plotted over *d*_norm_ in the range 0.3801 to +1.4738 a.u. is shown in Figure 8.

The overall two-dimensional ﬁngerprint plot is illustrated in Figure 8, and those are delineated into the major contacts: H H (62.6%), C H/H C (15.8%), O H/H O and (15.3%). The other contacts are negligible with individual contributions of less than 2.2% [N·· ·H/H·· ·N (2.2%), N·· ·C/C·· ·N (2.1%), C·· ·C (1.3%), and N·· ·C/C·· ·N (0.7%).

### Solid electronic spectra

**Fig. 9:**
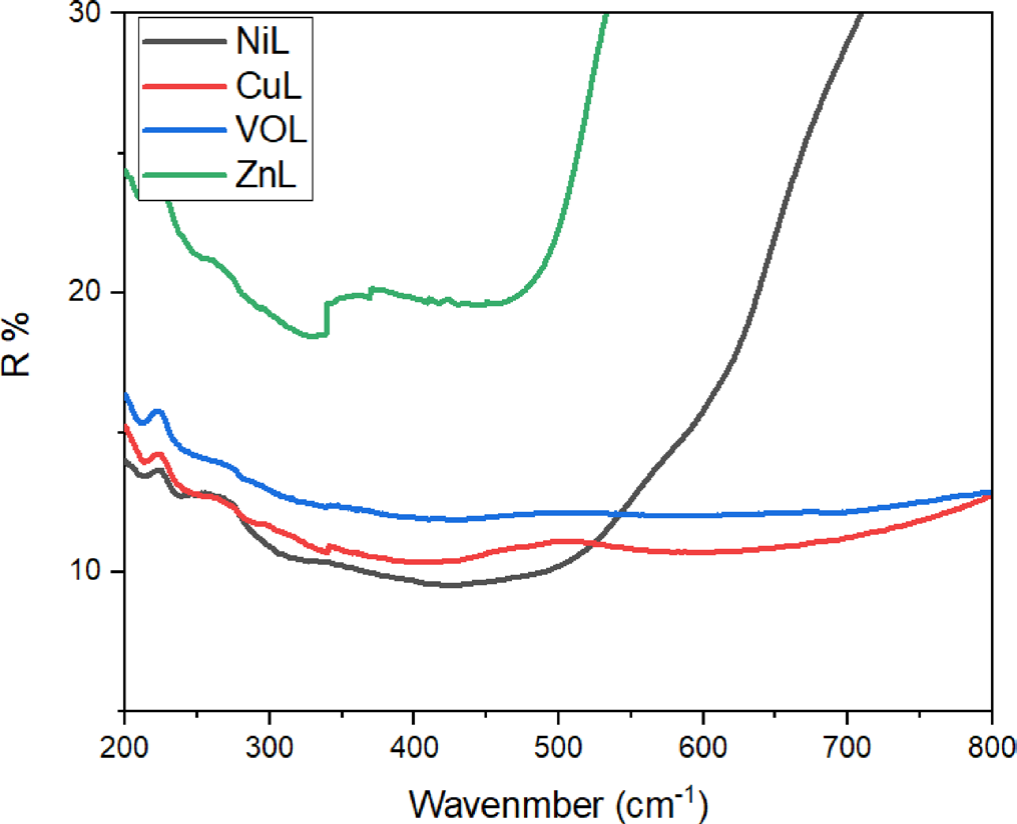
Diffuse reflectance electronic spectra at 298 K for complexes.

As illustrated in the diffuse reflectance electronic spectra (Fig. 9), the ligand field transition bands measured using the diffuse reflectance technique at 298 K are observed at 13,600 and 17,100 cm^−1^ for the ligand, and at 15,500 cm^−1^ for the complexes. These values are consistent with common ligand field transitions for tetrahedrally distorted planar or square planar chromophores, respectively. Additionally, continuous broad bands at 24,000 and 32,000 cm^−1^ can be attributed to ligand-to-metal charge transfer (LMCT) bands and π–π* transitions, respectively. The characteristics of the d–d bands suggest that distortion of the coordination environment from square planar to compressed tetrahedral affects the spin state of the ground state, with (S = 0) or (S = 1), respectively.

For the CuL complex, the electronic spectrum records broad bands at 13,900 and 16,660 cm^−1^, which are assigned to the transitions ^2^B_1g_ → ^2^B_2g_ and ^2^B_1g_ → ^2^E_g_ respectively. These transitions are specific to square-planar Cu^2^⁺ complexes. The magnetic moment was measured at approximately 2.08 BM, indicating the presence of one unpaired electron per Cu(II) ion in a square planar environment.

The electronic spectrum of the NiL complex displays two spin-allowed bands at 16,120 and 22,720 cm^−1^. These absorption bands can be assigned to the ^1^A_1g_ → ^1^A_2g_ and ^1^A_1g_ → ^1^E_g_ transitions, respectively, which are characteristic of d⁸ ions in a square planar geometrical environment. The ZnL complex was found to be diamagnetic, and an octahedral geometry was proposed based on its empirical formula, as shown in Table 1.

### Electron paramagnetic resonance (EPR)

**Fig. 10:**
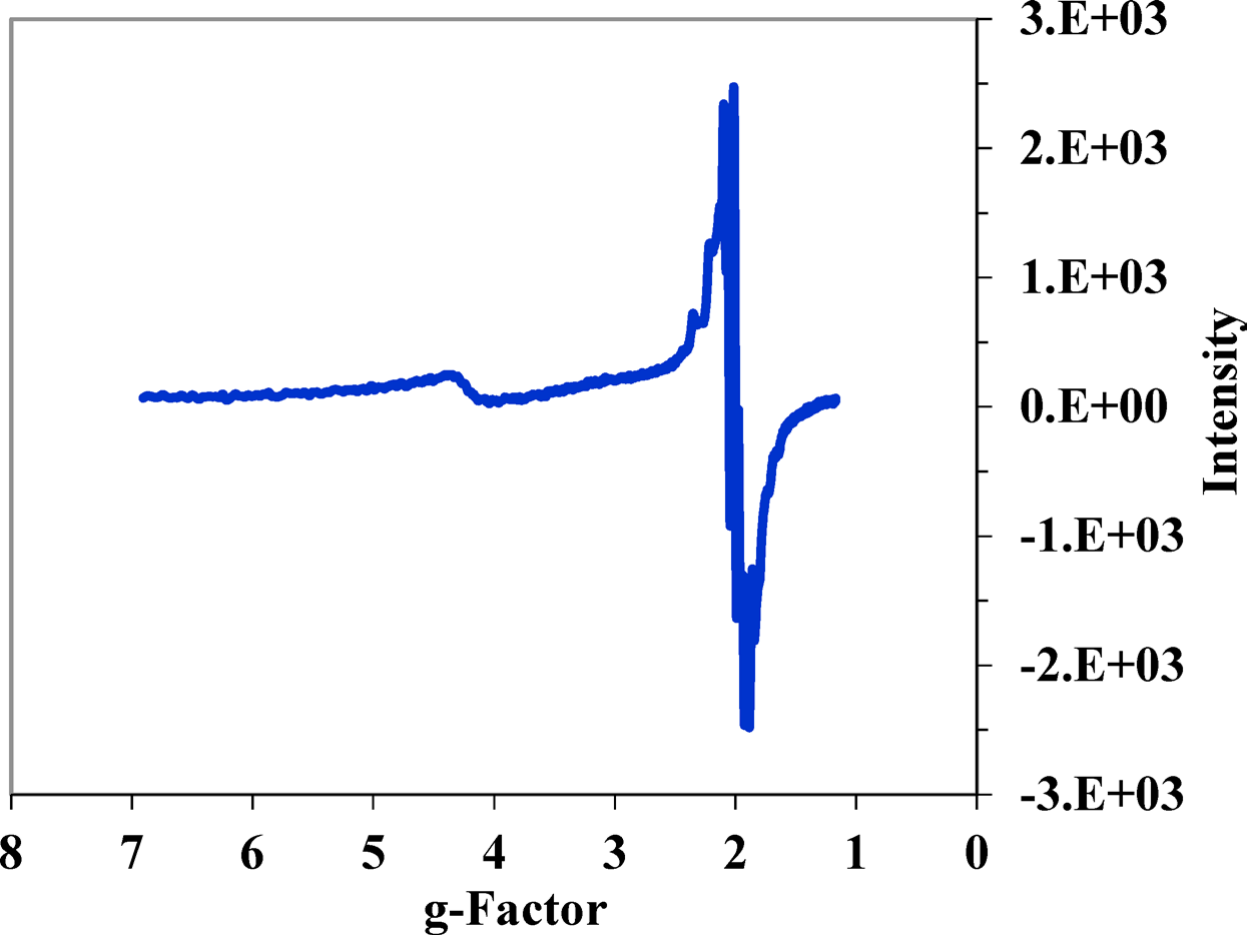
The X-band ESR spectrum of the complexes in DMSO.

The electron paramagnetic resonance (EPR) spectrum of the polycrystalline CuL complex was recorded at room temperature using an X-band frequency of 9.435 GHz. The EPR spectral data is presented in Fig. 10. The spin–orbit coupling of the ground state to excited states leads to Zeeman splitting, causing the g factors to deviate from the free-electron value of 2.0023. The data revealed that g// > g_┴_ > ge (2.0023), indicating that the copper(II) complexes exhibit either square planar or tetrahedral coordination geometry, with the single unpaired electron residing in the dx^2^-dy^2^ orbital.

According to the work of Garribba and Micera^48, the EPR spectrum of the CuL complex displayed a typical tetragonal Cu(II) environment, resulting in two g values: g parallel g // and g perpendicular g_┴,_ both of which are higher than the free electron g value. Our results align well with the expected characteristics of powdered EPR spectra for a series of bis(ethylenediamine) copper(II) complexes. When the ground state is associated with the d*x*^2^ − *y*^2^ or d*z*^2^ orbitals, the EPR spectra are axial, exhibiting equivalent x and y axes and presenting two g values: *g*// (*g*_*z*_) and *g*⊥ (*g*_*x*_ = *g*_*y*_). In cases where the geometry is elongated octahedral, square pyramidal, or square planar, the ground state is typically the d*x*^2^ − *y*^2^ orbital.

### Theoretical calculations

Computational technique for examining the electrical structure, geometries, and molecular characteristics of Schiff base metal complexes is a DFT analysis. By offering comprehensive insights into elements like charge distribution, energy gaps, and how the complexes will interact with biological targets via techniques like molecular docking, it enhances experimental findings. In addition to predicting biological activities, including antibacterial or antioxidant effects, of the ligand and its consequent metal complexes, this computational approach aids in the validation of experimental results. A variety of molecular parameters can be computed using DFT, such as bond lengths and bond ordering, charge distribution inside the molecule, dipole moment, and frontier molecular Frontier molecular orbitals (HOMO–LUMO gap), which relates to the molecule’s reactivity and optical properties. DFT is used to calculate a range of molecular properties, including: Bond lengths and bond orders, Charge distribution within the molecule, Dipole moment, Frontier molecular orbitals (HOMO–LUMO gap), which relates to the molecule’s reactivity and optical properties The computational results from DFT are used to Support and interpret experimental data, such as spectroscopy (IR, UV–Vis) and thermal analysis (TGA). Predict and explain biological activities, such as antimicrobial, anti-inflammatory, or antioxidant effects.

#### Molecular DFT calculation of ligand (HL)

**Fig. 11:**
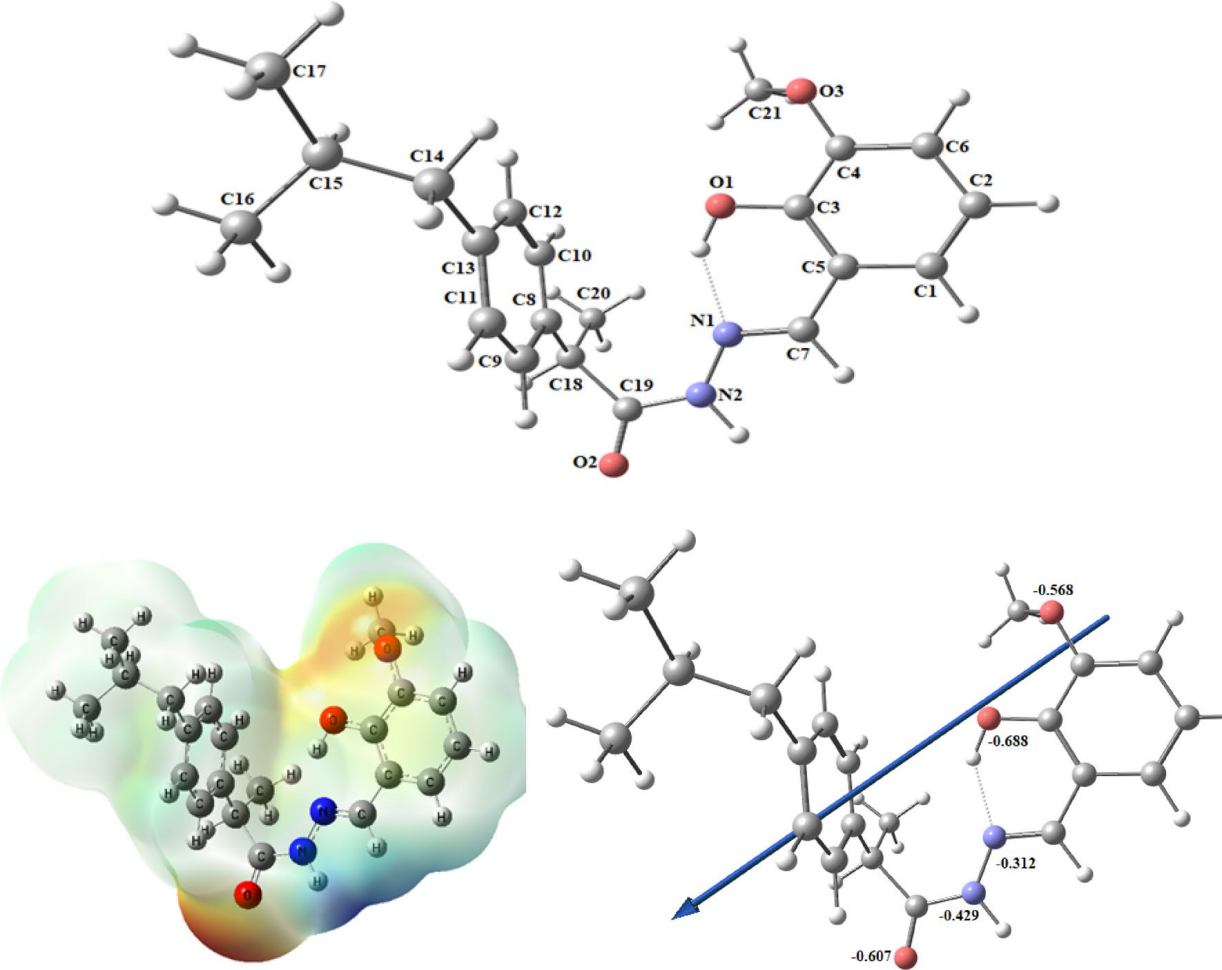
The optimized structure of the ligand, the vector of the dipole moment, the natural charges on atoms, and the molecular electrostatic potential (MEP) surface by density function B3LYP/6–311 +  + g(d, p).

Figure 11 shows the optimized structures of the ligand at the lowest energy configurations. The natural charges obtained from Natural Bond Orbital Analysis (NBO) show that the more negative active sites are O1 (-0.688), O2 (-0.607), O3 (-0.568), N1 (-0.212), and N2 (-0.429). The ligand is coordinated to metal ions as bidentate forming a 6-membered ring with O1 and N1 atoms.

#### Molecular DFT calculation of [CuL(H_2_O)Cl] and [NiL(H_2_O)NO_3_] complexes

**Fig. 12:**
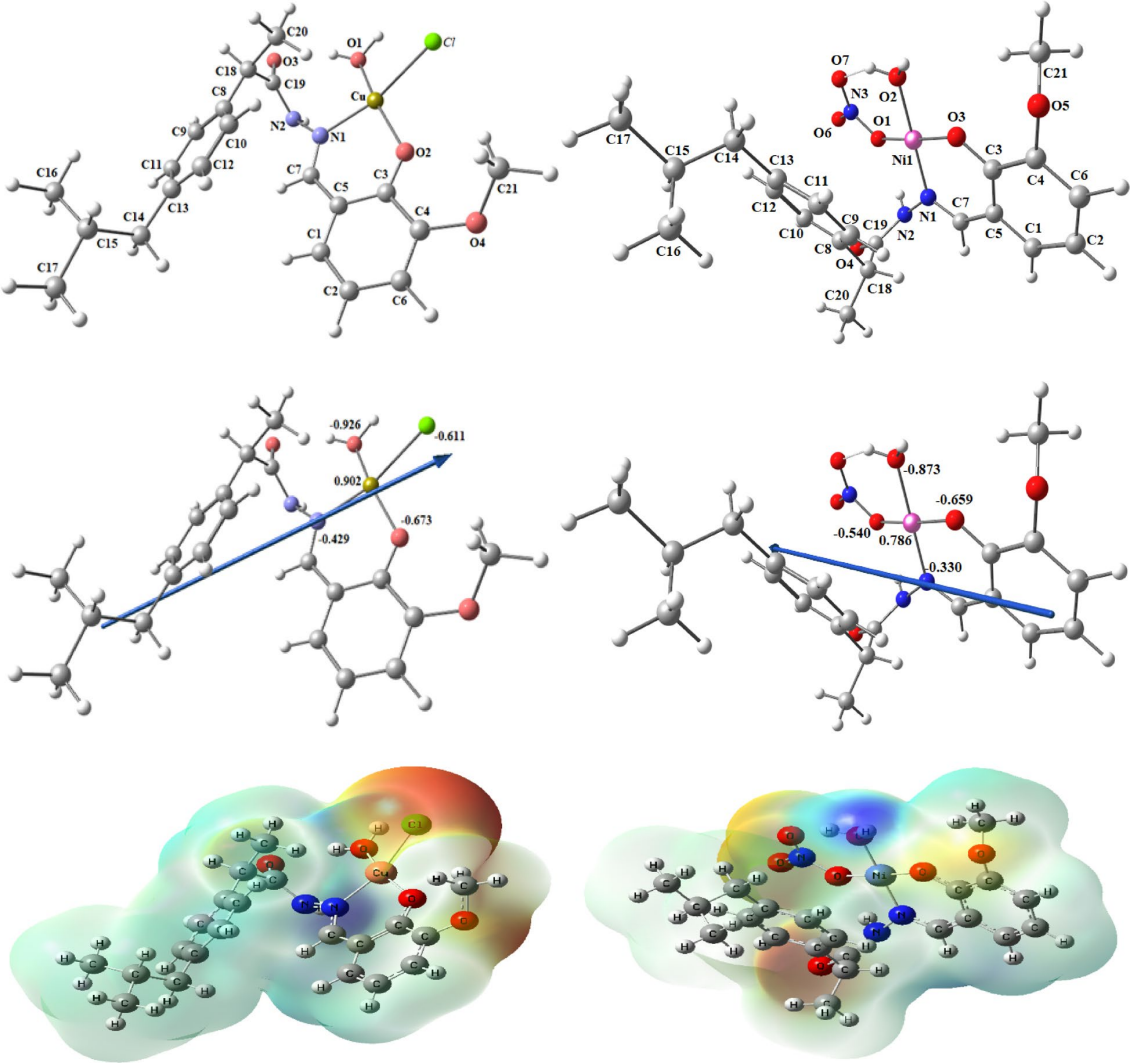
The optimized structure, the vector of the dipole moment, and the natural charges on active centers of [CuL(H_2_O)*Cl*] and [NiL(H_2_O)NO_3_] complexes.

Figure 12 shows the optimized structures of the complexes [CuL(H_2_O)*Cl*] and [NiL(H_2_O)NO_3_] as the lowest energy configurations. The copper and nickel atoms are four-coordinated in square planar geometries. The atoms N1, O1, *Cl,* and O2 in [CuL(H_2_O)*Cl*] are almost in one plane, deviated by -7.321°, and the atoms N1, O3, O2, and O1 in [NiL(H_2_O)NO_3_] are almost in one plane deviated by -3.059°, respectively, Table 4.

**Table 4:** Important optimized bond lengths (Å) and bond angles (°) of [CuL(H_2_O)*Cl*] and [NiL(H_2_O)NO_3_] complexes.

Bond lengths of [CuL(H_2_O)*Cl*]		Bond lengths of [NiL(H_2_O)NO_3_]	
Cu-O1 = 2.053	Cu-O2 = 1.911	Ni-O1 = 1.917	Ni-O2 = 1.897
Cu-N1 = 1.999	Cu-*Cl* = 2.333	Ni-N1 = 1.892	Ni-O3 = 1.842
Bond angles of [CuL(H_2_O)*Cl*]		Bond angles of [NiL(H_2_O)NO_3_]	
O1-Cu-O1 = 94.64	O1-Cu-O2 = 164.3	N1-Ni-O1 = 88.27	N1-Ni-O2 = 178.3
N1-Cu-O2 = 89.71	N1-Cu-*Cl* = 165.2	N1-Ni-O2 = 93.87	O1-Ni-O3 = 176.0
O1-Cu-*Cl* = 83.99		O1-Ni-O2 = 93.17	
O2-Cu-*Cl* = 96.57	N1-O1-*Cl*-O2 = -7.321*	O2-Ni-O3 = 84.73	N1-O3-O2-O1 = -3.059*

*Dihedral angle.

The natural charges computed from the NBO analysis on the coordinated atoms for [CuL(H_2_O)*Cl*] and [NiL(H_2_O)NO_3_] are [Cu (+ 0.884), N1 (-0.468), O1 (-0.990), O2 (-0.679) and *Cl* (-0.536)] and [Ni (+ 0.797), O1(-0.655), N1(-0.314), O2(-0.566) and O6(0.871)]; respectively.

#### DFT calculation of [VOL(H_2_O)OEt] and [ZnL(H_2_O)_3_NO_3_] complexes

**Fig. 13:**
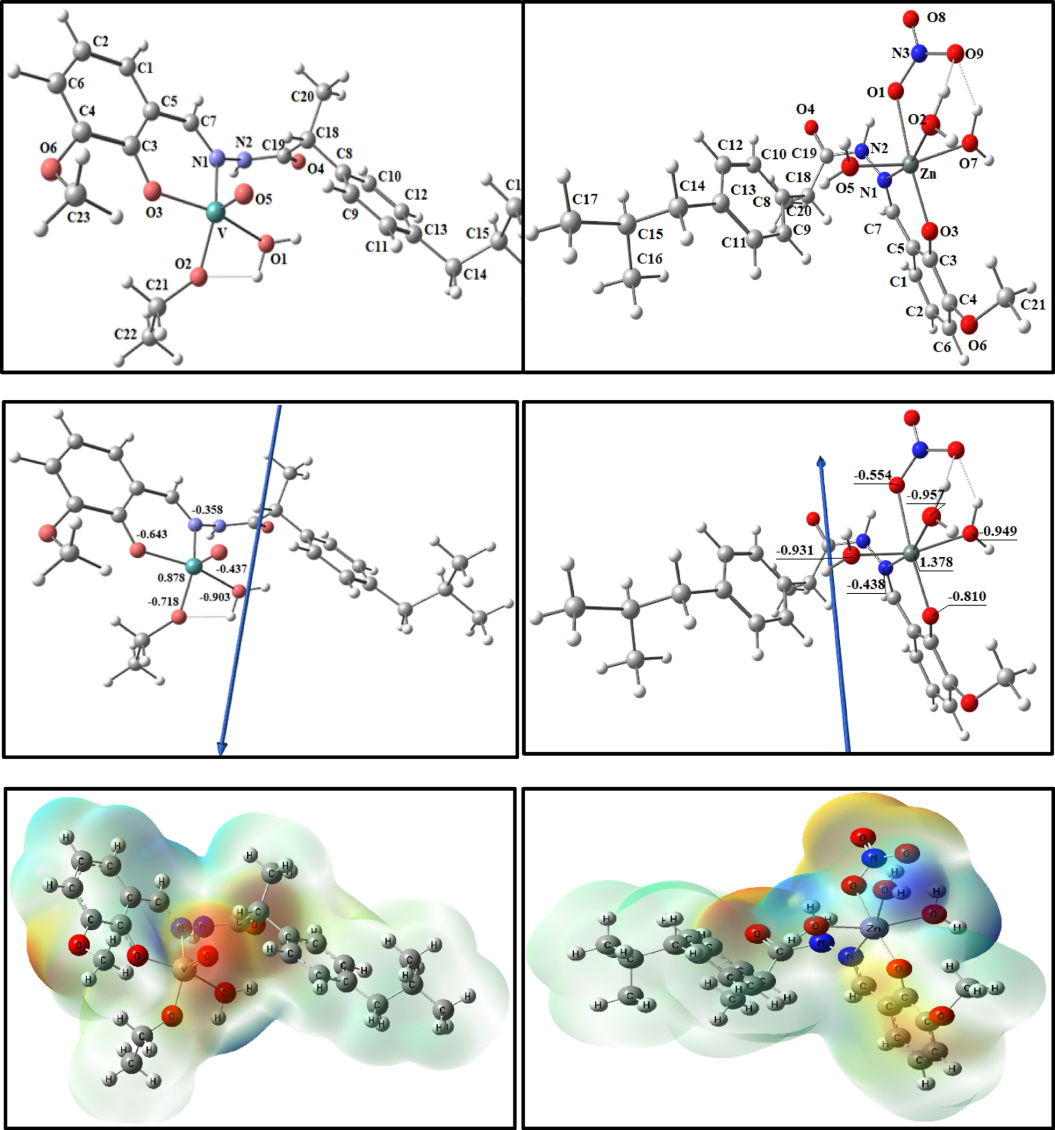
The optimized structure, the vector of the dipole moment, and the natural charges on active centers of [VOL(H_2_O)OEt] and [ZnL(H_2_O)_3_NO_3_] complexes.

Figure 13 and Table 5 show the optimized structures of the complexes [VOL(H_2_O)OEt] and [ZnL(H_2_O)_3_NO_3_] as the lowest energy configurations. The vanadium and zinc atoms are five- and six-coordinated in distorted square pyramidal and octahedral geometries, respectively.

**Table 5:** Important optimized bond lengths (Å) and bond angles (°) of [VOL(H_2_O)OEt] and [ZnL(H_2_O)_3_NO_3_] complexes [98]

Bond lengths of [VOL(H_2_O)OEt]		Bond angles of [VOL(H_2_O)OEt]	
V-N1 = 2.090	V-O3 = 1.934	N1-V-O1 = 90.60	O5-V-N1 = 108.1
V-O1 = 2.115	V = O5 = 1.612	N1-V-O3 = 85.74	O5-V-O1 = 97.51
V-O2 = 1.847		O1-V-O2 = 75.68	O5-V-O2 = 117.6
		O2-V-O3 = 91.82	O5-V-O3 = 103.0
Bond lengths of [ZnL(H_2_O)_3_NO_3_]		Bond lengths of [ZnL(H_2_O)_3_NO_3_]	
Zn-N1 = 2.139	Zn-O3 = 2.184	Zn-O2 = 2.162	Zn-O8 = 2.125
Zn-O1 = 2.032	Zn-O7 = 2.170		
Bond angles of [ZnL(H_2_O)_3_NO_3_]		Bond angles of [ZnL(H_2_O)_3_NO_3_]	
N1-Zn-O1 = 85.52	O3-Zn-N1 = 105.6	O8-Zn-N1 = 95.07	N1-Zn-O7 = 165.5
N1-Zn-O2 = 85.53	O3-Zn-O1 = 73.31	O8-Zn-O1 = 100.8	O1-Zn-O2 = 158.2
O1-Zn-O8 = 100.8	O3-Zn-O2 = 89.99	O8-Zn-O2 = 99.73	O2-Zn-O3 = 157.7
O2-Zn-O7 = 87.08	O3-Zn-O7 = 86.95	O8-Zn-O7 = 73.72	

The natural charges computed from the NBO-analysis on the coordinated atoms for [VOL(H_2_O)OEt] are V(+ 0.905), N1(-0.349), O1(-0.605), O2 (-0.724), O3 (-0.851) and O5(-0.427) and for [ZnL(H_2_O)_3_NO_3_] are Zn(+ 1.364), N1(-0.421), O1(-0.856), O2(-0.621), O3(-0.940), O7(-0.972) and O8(0.928).

**Fig. 14:**
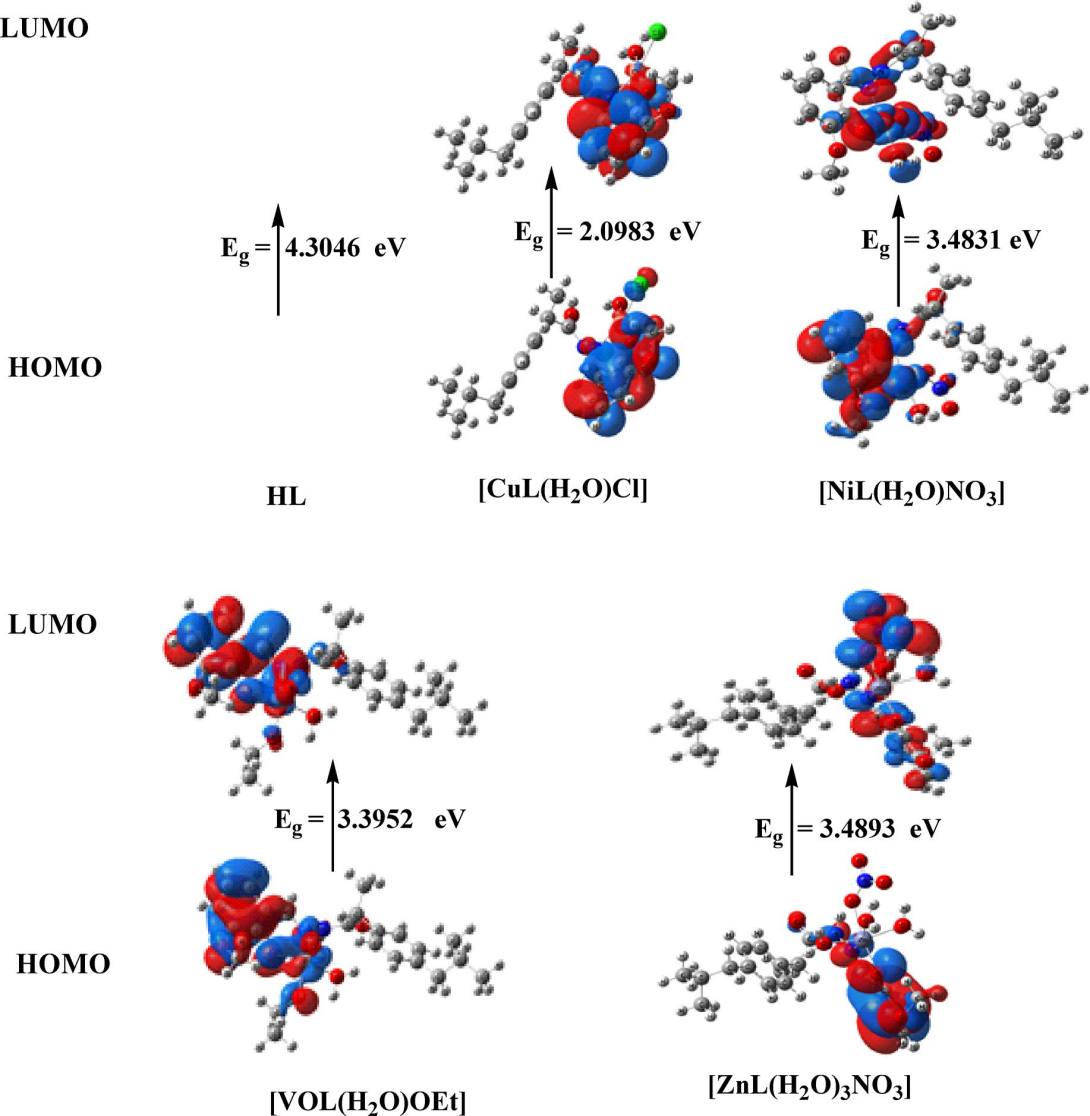
HOMO and LUMO charge density maps of HL ligand, [CuL(H_2_O)*Cl*_2_], [NiL(H_2_O)*Cl*_2_], [VOL(H_2_O)OEt], and [ZnL(H_2_O)NO_3_] complexes.

Figure 13 shows the MEP surface to locate the positive (blue color) and negative (red color, it is bound loosely or has excess electrons) charged electrostatic potential in the molecule. The computed total energy, the highest occupied molecular orbital (HOMO) energies, the lowest unoccupied molecular orbital (LUMO) energies, and the dipole moment for the ligands and complexes are presented in Table 6. The more negative values of the total energy of the complexes than that of the free ligand indicate that the complexes are more stable than the free ligand, and energy gap (E_g_) = E_LUMO_–E_HOMO_ is smaller in the case of complexes than that of the ligand due to chelation of the ligand to metal ions, Table 6. The lowering of E_g_ in complexes compared to the ligands explains the charge transfer interactions upon complex formation (Fig. 14).

**Table 6:** Calculated energies of ligand, [CuL(H_2_O)*Cl*_2_], [NiL(H_2_O)*Cl*_2_], [VOL(H_2_O)OEt] and [ZnL(H_2_O)_3_NO_3_ ] complexes.

	E ^a^	HOMO ^b^	LUMO ^c^	E_g_^d^	Dipole moment ^e^
HL	-1151.369	-6.2500	-1.9454	4.3046	2.3594
[CuL(H_2_O)*Cl*]	-1883.292	-5.9729	-3.8746	2.0983	8.0952
[NiL(H_2_O)NO_3_]	-1676.525	-6.3381	-2.8550	3.4831	3.3776
[VOL(H_2_O)OEt]	-1527.886	-6.0821	-2.6869	3.3952	1.3773
[ZnL(H_2_O)_3_NO_3_]	-1764.863	-5.5032	-2.0139	3.4893	4.2750

^a^E: the total energy (a.u.). ^b^HOMO: highest occupied molecular orbital (eV).

^c^LUMO: lowest unoccupied molecular orbital (eV).

^d^E_g_ = E_LUMO_- E_HOMO_ (eV). ^e^ dipole moment (Debye).

#### Reactivity studies

Many reactivity descriptors, such as ionization potential (I), electron affinity (A), electronegativity (χ), chemical potential (μ), hardness (η), softness (S), and electrophilicity index (ω), all derived from the HOMO and LUMO energies, have been proposed to understand various aspects of reactivity associated with chemical reactions (Table 7). This trend suggests that metal complexes exhibit higher polarizability and potential reactivity compared to the free ligand. The chemical potential (μ) became more negative, changing from -4.0977 eV for the ligand to -4.9237 eV for the Cu(II) complex, indicating a greater tendency of the complex to lose electrons.

**Table 7:** The ionization energy, I, electron affinity, A, electronegativity, χ, global softness, S, chemical hardness, η, and chemical potential, μ, were calculated for the ligand and complexes.

	ionization potential I = -E_HOMO_	electron affinity A = -E_LUMO_	Electro-negativity χ = (I + A)/2	chemical hardness η = (I -A)/2	chemical softness S = 1/2η	chemical potential μ = -χ	Electrophilicity ω = μ^2^/2η
HL	6.2500	1.9454	4.0977	2.1523	0.2323	-4.0977	3.9007
[CuL(H_2_O)Cl]	5.9729	3.8746	4.9237	1.0491	0.4766	-4.9237	11.5538
[NiL(H_2_O)NO_3_]	6.3381	2.855	4.5965	1.7415	0.2871	-4.5965	6.0660
[VOL(H_2_O)OEt]	6.0821	2.6869	4.3845	1.6976	0.2945	-4.3845	5.6621
[ZnL(H_2_O)_3_NO_3_]	5.5032	2.0139	3.7585	1.7446	0.2866	-3.7585	4.0486

In quantum chemistry, the HOMO and LUMO frontier orbitals in the molecular orbital diagram of a chemical compound are crucial, as they play a significant role in predicting the electric and optical properties of a molecule and its interactions with other species. The energy gap ((E_g)), defined as the energy difference between (E_{HOMO}) and (E_{LUMO}), is an important factor; the smaller the energy gap, the more reactive the complex. Thus, the order of increasing reactivity is as follows: [CuL(H₂O)Cl] > [VOL(H₂O)OEt] > [NiL(H₂O)NO₃] > [ZnL(H₂O)₃NO₃] > HL. A greater energy gap (ΔE) indicates that the compound is harder, while a smaller energy gap suggests easier charge transfer and polarization within the molecule. Therefore, the results indicate that a smaller energy gap corresponds to a softer compound.

The (E-{HOMO}) values reflect the donating properties (ionization potential) of the compounds, following the order: HL > [ZnL(H₂O)₃NO₃] > [NiL(H₂O)NO₃] > [VOL(H₂O)OEt] > [CuL(H₂O)Cl].

### Antimicrobial activity of the ligand and its metal complexes

The free HL ligand and CuL, NiL, VOL and ZnL complexes were tested against bacterial and fungal species by measuring the size of the bacteriostatic diameter. Table 8 shows the results, which reveal that the chemicals tested exhibit strong bactericidal and fungicidal activity against a wide range of bacteria and fungi.^33,34^.

**Table 8:** Results of activity index (%) for anti-bacterial assay of the prepared ibuprofen Schiff base ligands HL and its metal complexes.

Compound	Activity index (%)			
	S. aureus (+ ve)	B. subtilis (+ ve)	E. coli (-ve)	P. vulgaris (-ve)
CuL	41.67	80.77	40	56
NiL	37.5	80.77	0	48
ZnL	0	53.85	0	40
VOL	0	46.15	0	0
HL	0	38.46	0	0

#### Antibacterial activity

In vitro tests were conducted on synthetic compounds and the conventional antibiotic Gentamycin against two Gram-positive bacteria *(Staphylococcus aureus* and *Bacillus subtilis)* and two Gram-negative bacteria *(Escherichia coli and Proteus vulgaris).* The antibacterial activity of the compounds was assessed using the agar well diffusion technique, with the results presented in Table 8. The mononuclear metal complexes demonstrated greater antibacterial efficacy than the free Schiff base (HL). Additionally, it was established that the metal complexes exhibited more antipathogenic activity against Gram-positive bacteria compared to Gram-negative bacteria, which can be attributed to the differences in the complexity of the cell walls of these bacteria.

The results indicate that the Schiff base (HL) had the least effect against *B. subtilis, S. aureus, E. coli, and P. vulgaris.* The CuL complex exhibited strong antibacterial efficacy against all tested bacteria, including *B. subtilis, S. aureus, E. coli, and P. vulgaris.* The NiL complex showed high efficacy primarily against *S. aureus, B. subtilis,* and *P. vulgaris,* while the ZnL complex demonstrated efficacy only against *B. subtilis* and *P. vulgaris.* The VOL complex showed limited activity against* B. subtilis.*

**Fig. 15:**
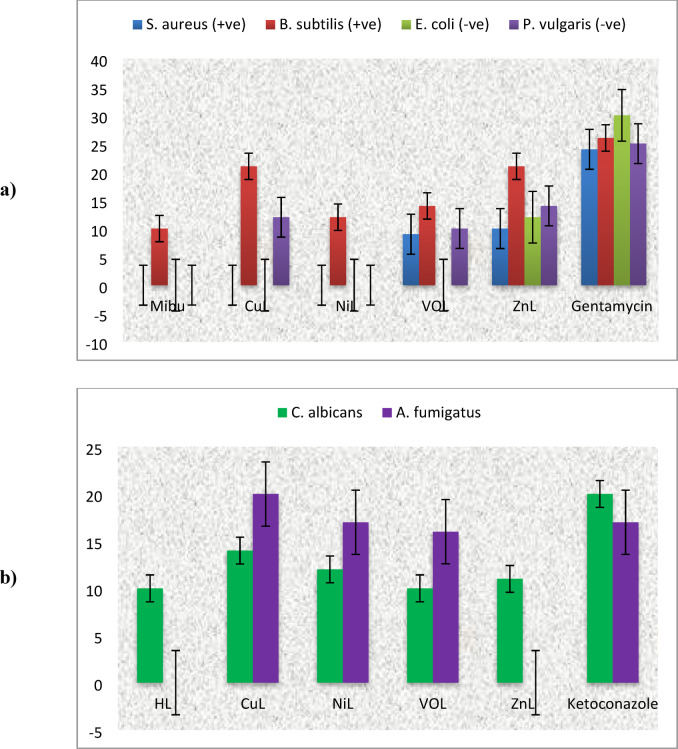
Inhibition zone and their standard deviation values of HL ligand and its complexes for bacteria and fungi.

The varying antibacterial effects of the different metal complexes against various microbes may be due to factors such as cell impermeability or ribosomal differences within the microbial cells. As illustrated in Fig. 15 and Figure S8, the lipid membrane surrounding the cell wall allows many lipophilic compounds to penetrate, highlighting that liposolubility is a fundamental characteristic in antimicrobial action.

Table 8 and Eq. 4 show how to calculate the activity index percentage^35–39^. The order of antibacterial activity is as follows: CuL > NiL > ZnL > VOL. By comparing these results with previous work, it was found that the presence of the methoxy group is more effective than the phenyl group, leading to a higher activity index for the CuL complex in this study compared to the CuL complex reported previously.

4 $${\mathrm{Activity}}\;{\mathrm{index}}\left( {{\% }} \right) = \frac{{Inhibition\;zone\;of\;compound\left( {{\mathrm{mm}}} \right)}}{{Inhibition\;zone\;of\;standard\;drug\left( {{\mathrm{mm}}} \right)}} \times 100$$

#### Antifungal activity

The new Schiff base (HL) and its metal complexes were tested in vitro against two fungi, *Candida albicans* and *Aspergillus fumigatus*, with results compared to Ketoconazole, a commonly used antifungal drug. The findings indicate that the metal complexes exhibit greater antifungal activity than the Schiff base (HL) ligand, as shown in Table 8. Among the tested compounds, the CuL complex was the most effective against all fungi, particularly against *A. fumigatus*. The DMSO control exhibited no antifungal activity.

All metal complexes demonstrated good antifungal activity against C. albicans and A. fumigatus, except for the HL ligand and VOL complex, which showed no activity against *A. fumigatus*. The antifungal activity of the complexes is strongly correlated with the type of metal ion present, with CuL complexes displaying the strongest impact, followed by VOL compounds and HL ligands. The lower activity observed for some metal complexes in this study may be attributed to their low lipophilicity, which hinders their ability to penetrate the lipid membrane and effectively suppress microbial growth.

The higher activity of metal chelates can be explained using chelation theory. During chelation, ligand orbital overlaps and partial sharing of the metal ion’s positive charge with the donor group reduce the polarity of the metal ion. This enhancement facilitates the penetration of complexes into lipid membranes and blocks metal binding sites in microbial enzymes. Furthermore, these complexes can inhibit the respiration mechanism of the cell nucleus, blocking protein synthesis and thereby restricting microbial growth.

The biological activity of the compounds is influenced by several factors, including the type of ligand, concentration, nature of metal ions, the nature of the anion surrounding the metal ion, coordinating sites, and the form of the complexes. The VOL complex exhibited lower antibacterial activity than other synthesized metal complexes, which may be due to the inhibition mechanism associated with its altered square pyramidal structure. As illustrated in Figs. 15 and S8^26,38^, the order of antifungal activity is as follows: CuL > NiL > ZnL > VOL. Notably, the CuL complexes in this study demonstrated a larger inhibition zone compared to the CuL complex reported in previous research due to the methoxy group.

### Cytotoxic activity evaluation for the new compounds

Furthermore, the new complexes demonstrated greater susceptibility to cytotoxicity against HepG-2, MCF-7, and HCT-116 compared to the other cancer cells. Notably, the CuL complex outperformed the other new complexes and the Schiff base ligands in cytotoxicity tests against all three cell types. Its IC₅₀ values against the three cancer cell lines were very comparable to those of vinblastine: CuL had IC₅₀ values of 5.07 μM, 6.54 μM, and 4.39 μM against MCF-7, HCT-116, and HepG-2, respectively, while vinblastine had IC₅₀ values of 4.46 μM, 5.25 μM, and 4.10 μM for the same cell lines.

**Fig. 16:**
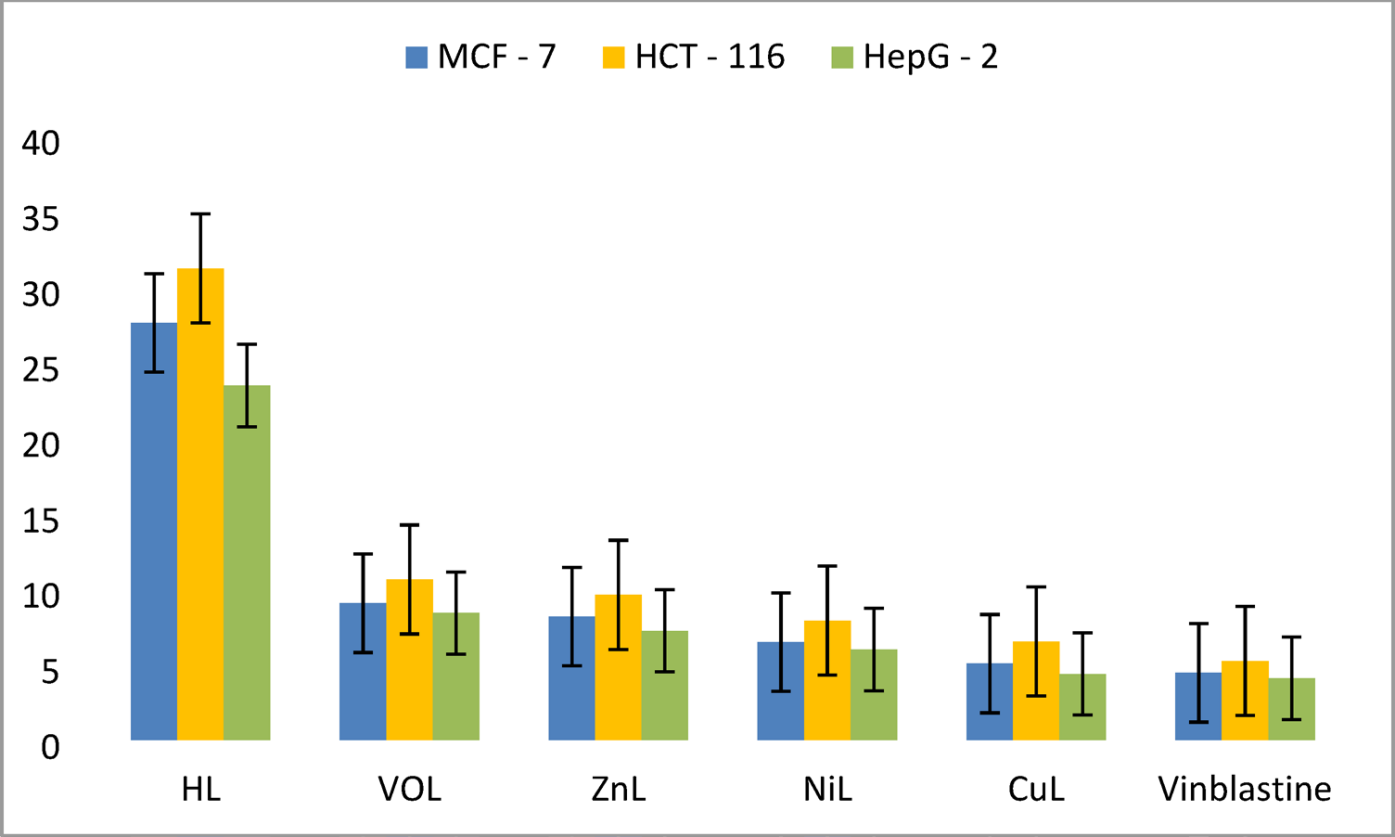
IC_50_ values of HL ligand and its complexes against breast cancer cell line MCF-7, colon cancer cell line HCT-116 and liver cancer cell line HepG-2 compared to Vinblastine.

These findings demonstrate that CuL is the most active anticancer agent among the tested compounds, particularly against the breast cancer cell line MCF-7. The order of activity for the new complexes is as follows: CuL > NiL > ZnL > VOL > HL, as illustrated in Fig. 16^30,40^. The cytotoxicity results of our new compounds, derived from the Schiff base ligand of Ibuprofen with a methoxy side chain, revealed that these new compounds possess higher activity and lower IC₅₀ values compared to previously reported ibuprofen Schiff base complexes, which contained an aromatic side chain.

### CT-DNA binding of HL and its complexes

#### Spectral method

The most effective method for determining the mode of interaction or binding potential of key small compounds with CT-DNA is spectrophotometric titration. A strong interaction between the nitrogen bases of DNA and the coordinating ligand can be indicated by hypochromic or bathochromic shifts, reflecting complex interactions. Depending on the metal charge, chelate structure, and nature, the complexes may interact with double-stranded DNA.

When a metal-specific chelate interacts with DNA, the absorption bands of the chelate undergo changes. The degree of interaction can be related to the extent of the absorption change and/or the position of this change. In cases of covalent binding, a secondary ligand (labile ligand), such as the H₂O ligand in a metal complex, could be replaced by a nitrogen base of DNA, such as guanine N7. Additionally, electrostatic interactions with the minor and major grooves of DNA suggest non-covalent contacts.

The π* orbital of intercalated ligands or complexes may overlap with the orbitals of base pairs in CT-DNA in an intercalative mode, leading to a decrease in the transition energy of π → π* transitions and resulting in hypochromicity^41^. The binding capability of the CuL, NiL, ZnL, and VOL complexes with CT-DNA was assessed by observing changes in their electronic spectra following stepwise DNA addition.

**Fig. 17:**
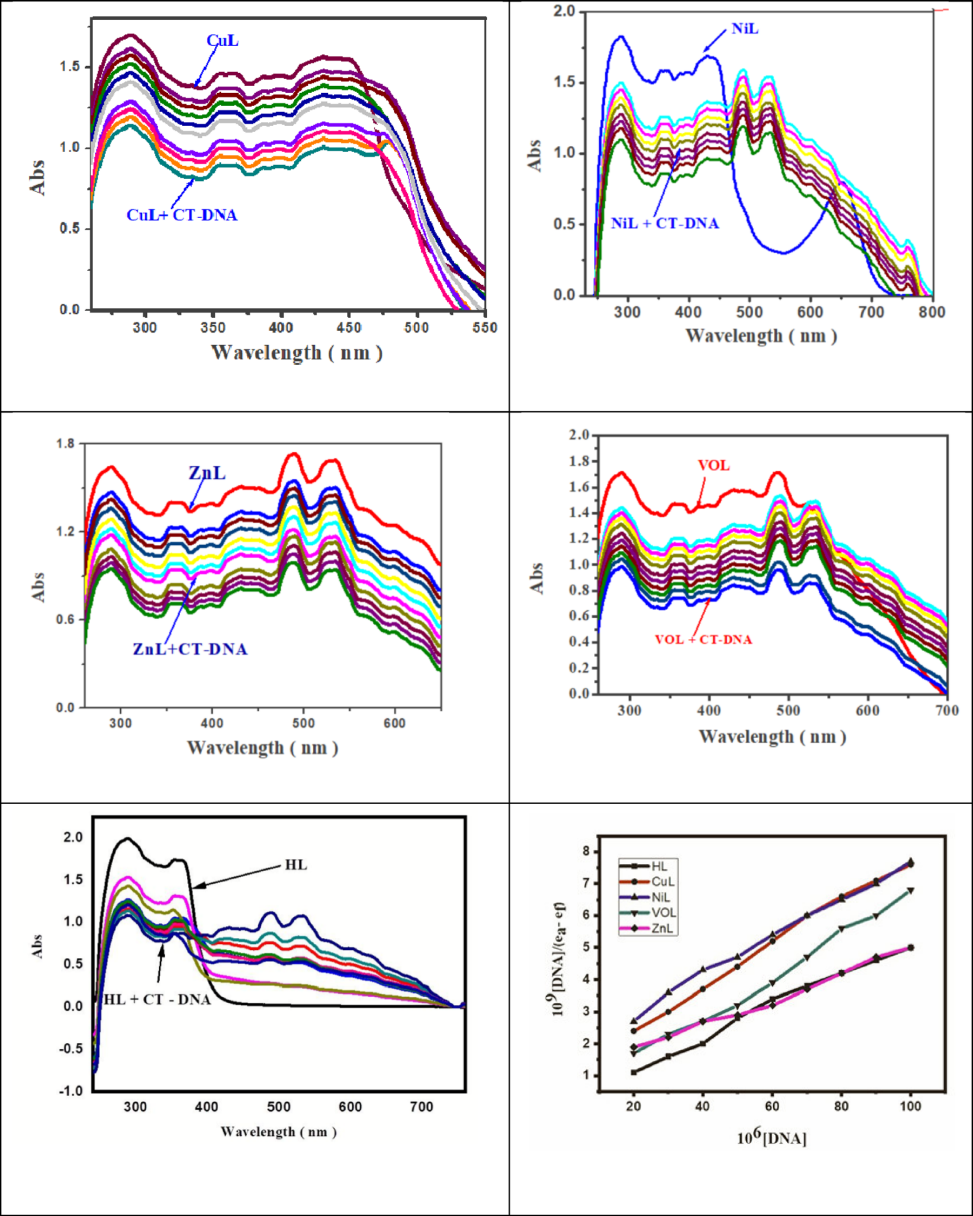
Absorption spectra of the complexes a) CuL, b) NiL, c) ZnL, d) VOL e) HL ligand (in Tris–HCl/NaCl buffer) on addition of increasing concentration of CT-DNA. [complex] = 10 μM, [DNA] = 10–100 μM, and shows changes in absorbance upon increasing amounts of CT-DNA. Plot of [DNA]/(ε_a_ − ε_f_) versus [DNA] for the titration of DNA with metal complexes.

As shown in Table 9, the absorption bands of the examined complex (CuL) changed as the DNA content increased, indicating a hypochromic effect caused by interaction with CT-DNA. This hypochromic change further suggests that DNA interacts with electropositive substances through electrostatic forces. The calculated binding constants (Kb) for the complexes were found in the following order: CuL > NiL > ZnL > VOL, as illustrated in Fig. 17^42–44^.

**Table 9:** Spectral parameters for the interaction of CT- DNA with the new complexes.

Compound	${\lambda }_{max}$ *nm* , Free	${\lambda }_{max}$ *nm*, Bonded	$\Delta n$	K_b_ $\times {10}^{6}$	Chromism %	Type of Chromism	$\Delta G$ *KJ / mol*
CuL	289	288	1	6.5	4.73	Hypo	-33.16
	360	361	1		5.52		
	430	428	2		2		
NiL	290	291	1	4.8	18.58	Hypo	-32.41
	359	360	1		20.25		
	431	430	1		18.93		
ZnL	289	287	2	4	10.37	Hypo	-31.96
	359	357	2		11.51		
	430	431	1		11.26		
	488	489	1		10.40		
	533	534	1		11.24		
VOL	290	289	1	3.5	15.79	Hypo	-31.63
	360	361	1		19.05		
	431	429	2		17.09		
	485	488	3		10.53		
HL	288	289	1	2.4	5.92	Hypo	-30.69
	355	354	1		4.86

#### Gel electrophoresis

Gel electrophoresis is a standard method for assessing the binding of compounds to nucleic acids. In this procedure, molecules are separated based on their relative rates of movement through a gel under the influence of an electric field. When DNA is subjected to an electric field, it migrates toward the anode due to its negative charge. The extent of migration is influenced by several factors, including the strength of the electric field, the buffer used, the density of the agarose gel, and the size of the DNA. It is generally assumed that DNA’s mobility is inversely proportional to its size.

Photographs of the gel reveal bands with varying widths and brightness when compared to the control. The interaction of the new complexes with DNA was examined using agarose gel electrophoresis, and the results are presented in Figure S9. The DNA-cleavage effectiveness of the investigated complexes can be attributed to their binding affinity for DNA.

The results indicate that the complexes can be arranged according to their DNA cleavage potency in the following order: CuL > NiL > ZnL > VOL > HL, with a corresponding decrease in brightness of the lanes. These findings align with the binding constant values of the CT-DNA complexes presented in Table 9^45^. Comparing these results to previous work, it was found that the complexes of the Schiff base ligand with the methoxy side chain are more effective than those with the phenyl group reported earlier.

### The mechanism of action for metal complexes used as antibacterial agents

Tweedy’s chelation theory proposes that when metal ions bind to ligands, the resulting coordination reduces the polarity of the metal atoms. This occurs through two mechanisms: delocalization of π-electrons across the entire chelate structure and partial transfer of positive charge to the donor groups. The diminished polarity enhances the lipophilicity of these complexes, thereby improving their ability to penetrate bacterial lipid membranes. Following chelation, the altered properties of the metal complexes—including electronic configuration and charge distribution—may potentiate their biological activity. Specifically, the metal ion’s decreased polarity arises from orbital overlap with ligands and charge-sharing with donor atoms. These modifications facilitate the complex’s passage through hydrophobic cellular barriers, enabling it to form stable covalent bonds within bacterial enzymes. Such binding effectively blocks essential metal-coordinating sites, disrupting enzymatic function.

### In vitro anti-inflammatory activity

Inflammation is a biologically defensive response triggered by various stresses, including heat, radiation, microbial infections, and chemical or physical agents, and it is often associated with pain. Arachidonic acid metabolism plays a significant role in the mechanism of inflammation through the production of prostaglandins via the cyclooxygenase-2 (COX-2) enzyme cascade. Inhibition of COX-2 can directly reduce prostaglandin production^46^. These hormones, known as prostaglandins, prompt the body to react to painful stimuli. Consequently, the development of an anti-inflammatory and analgesic drug capable of blocking these enzymes is of considerable interest.

**Fig. 18:**
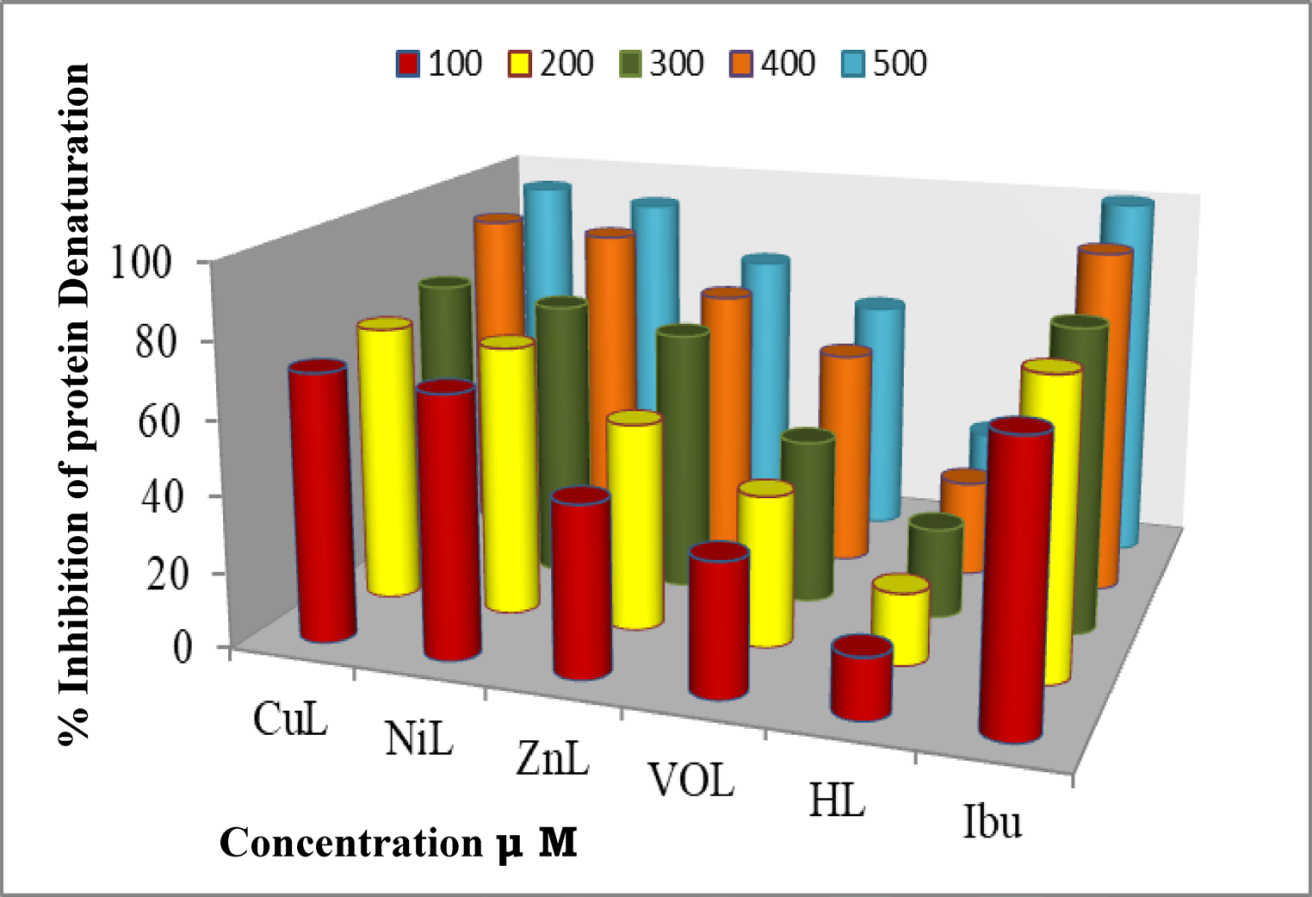
Percent of inhibition of protein denaturation of the HL ligand and its metal complexes compared to ibuprofen at different concentrations.

The percentage of inhibition of protein denaturation in fresh egg albumin was measured to evaluate the anti-inflammatory potency of the new complexes and the reference medication ibuprofen. The data are presented in Fig. 18 and Table S4. The anti-inflammatory results indicate that as the concentration of the complexes increases, the degree of protein denaturation decreases. Compared to ibuprofen, the ligand and its new complexes demonstrate significant anti-inflammatory efficacy. The anti-inflammatory potency of the metal complexes was markedly higher than that of the free ligand.

The enhanced activity of the metal complexes can be explained using chelation theory. Among the tested compounds, CuL emerges as a promising anti-inflammatory agent that could be utilized to treat inflammation-related conditions. The order of inhibition of protein denaturation is as follows: CuL > NiL > ZnL > VOL, with CuL being the most effective anti-inflammatory metal complex, as shown in Fig. 18^47^.

Furthermore, when comparing the results with previous work, it was found that the inhibition of protein denaturation by CuL with the second ligand is more effective than that of CuL with the first ligand, attributed to the presence of the methoxy group.

### Docking investigation

#### The docking of the ligand and its complexes agaist SARS- COVID-19 main protease

**Fig. 19:**
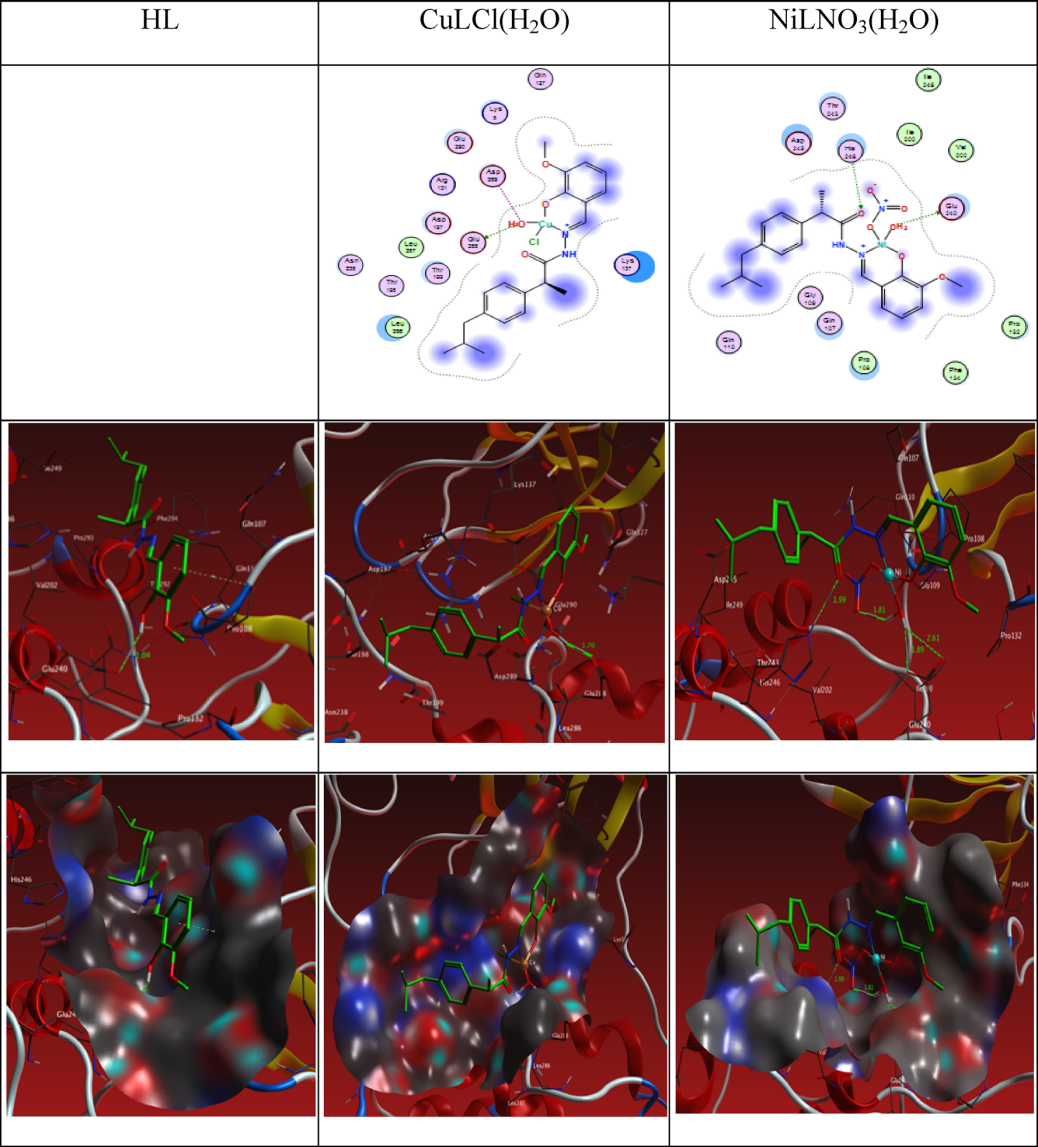
2D and 3D plots of the interaction between HL, CuLCl(H_2_O), and NiLNO_3_(H_2_O) with the active site of the receptor of viral protein (PDB ID: 6lu7). Hydrophobic interactions with amino acid residues are shown with dotted curves.

Over the past ten years, computer-aided drug discovery (CADD) has been utilized to investigate drug-target interactions because the process of getting a candidate medicine to clinical use is extremely expensive. We chose to conduct a docking-based virtual screening in order to determine the compounds’ possible inhibitory action against SARS- COVID-19 main protease (6LU7), taking advantage of the crystal structure of the enzyme being available at the Worldwide Protein Data Bank. The molecular docking and 2D and 3Ddiagram of the interactions between the Schiff base ligand HL and the its complexes CuL, NiL, VOL and ZnL with 6LU7 are shown in Fig. 19 and the binding energy values are shown in Table 10. In HL ligand, two hydrogen bonding interactions were observed between H of the ligand to O7 of OE1 GLU 240 and pi-sigma interaction between a ring of the ligand to CD PRO 108 at distance of 2.93 and 4.09 Å as shown in figure in Table 10 and Fig. 19

**Table 10:** The Docking interaction data calculations of HL, CuLCl(H_2_O), NiLNO_3_(H_2_O), VOL(OEt)(H_2_O), and ZnLNO_3_(H_2_O)_3_ with the active sites of the COVID-19 receptor main protease viral protein (PDB ID: 6lu7).

	Receptor	Interaction	Distance(Å)*	E (kcal/mol)
HL				
O 7	OE1 GLU 240	H-donor	2.93 (2.04)	-3.8
6-ring	CD PRO 108	pi-H	4.09	-0.6
CuLCl(H_2_O)				
O 2	OE1 GLU 288	H-donor	2.70 (1.70)	-15.5
O 2	OE1 GLU 288	Ionic	2.70	-6.9
O 2	OD1 ASP 289	Ionic	3.54	-1.8
NiLNO_3_(H_2_O)				
	OE1 GLU 240	H-donor	3.43 (2.61)	-0.7
O 2	OE2 GLU 240	H-donor	2.72 (1.89)	-15.2
O 27	NE2 HIS 246	H-acceptor	2.98 (1.99)	-4.3
O 2	OE1 GLU 240	Ionic	3.43	-2.2
O 2	OE2 GLU 240	Ionic	2.72	-6.6
VOL(OEt)(H_2_O)				
O 29	NZ LYS 269	H-acceptor	2.91 (1.91)	-10.4
N 12	OD2 ASP 229	Ionic	2.87	-5.4
O 29	NZ LYS 269	Ionic	2.91	-5.1
ZnLNO_3_(H_2_O)_3_				
O 2	OE1 GLU 288	H-donor	2.92 (2.00)	-13.6
O 59	OD1 ASP 289	H-donor	2.61 (1.61)	-27.9
O 2	OE1 GLU 288	Ionic	2.92	-5.1
O 2	OD1 ASP 289	Ionic	3.23	-3.1
O 28	OE1 GLU 288	Ionic	3.48	-2.0
O 59	OD1 ASP 289	Ionic	2.61	-7.6
O 59	OE2 GLU 290	Ionic	3.32	-2.6
6-ring	NZ LYS 137	pi-cation	3.21	-0.8

*The lengths of H-bonds are in brackets.

In the CuL complex, one H-bonds between O2 the ligand to O of OE1 GLU 288 at 2.70 Å. Two ionic bonds between O2 of the ligand to OE1 GLU 288 and OD1 ASP 289 at 2.70 and 3.54 Å respectively. Five interactions between the NiL Complex and the residues of the active sites of the receptor of the main protease viral protein 6LU7 two H-donor between O2 and OE1 GLU 240 and OE2 GLU 240 at 3.43 and 2.72 Å, one H-acceptor between O2 and NE2 HIS 246 at 2.98 Å and two ionic bonds between O2 and OE1 GLU 240 and OE2 GLU 240 at 3.43 and 2.72 Å.

Three interactions were found between the VOL complex and the main protease viral protein 6LU7, one H-acceptor between O29 and NZ LYS 269 at 2.91 Å. Two ionic bonds between N 12 and O29 and OD2 ASP 229 and NZ LYS 269 at 2.87 and 2.91 respectively. Furthermore, the ZnL complex has shown eight interactions, two hydrogen bonds between O2 and O59 of ligand to OE1 GLU 288 and OD1 ASP 289 were found at 2.91, and 2.61 Å. Five ionic bonds were seen between O2, O2, O28, O59, O59 of the Schiff base and OD1 ASP 289, OE1 GLU 288, OD1 ASP 289 , OE1 GLU 288 , OD1 ASP 289 OE2 GLU 290 were found at 2.92, 3.23, 3.48, 2.61, and 3.32 Å. One pi-cation interaction between 6 ring of ligand to NZ LYS 137 at 3.21 Å.

The binding free energy of ligand and its complexes with the receptor of COVID-19 main protease viral protein (PDB ID: 6lu7) are found to be -4.4, -24.2, -29.0, -20.9 and -62.7 kcal/mol for HL, CuLCl(H_2_O), NiLNO_3_(H_2_O), VOL(OEt)(H_2_O) and ZnLNO_3_(H_2_O)_3_; respectively, Table 10. The more negative the binding energy, the stronger the interaction. So, the interactions are in the order of HL ˂VOL(OEt)(H_2_O) ˂ ZnLNO_3_(H_2_O)_3_ ˂ NiLNO_3_(H_2_O) ˂ CuLCl(H_2_O).

**Fig. 20:**
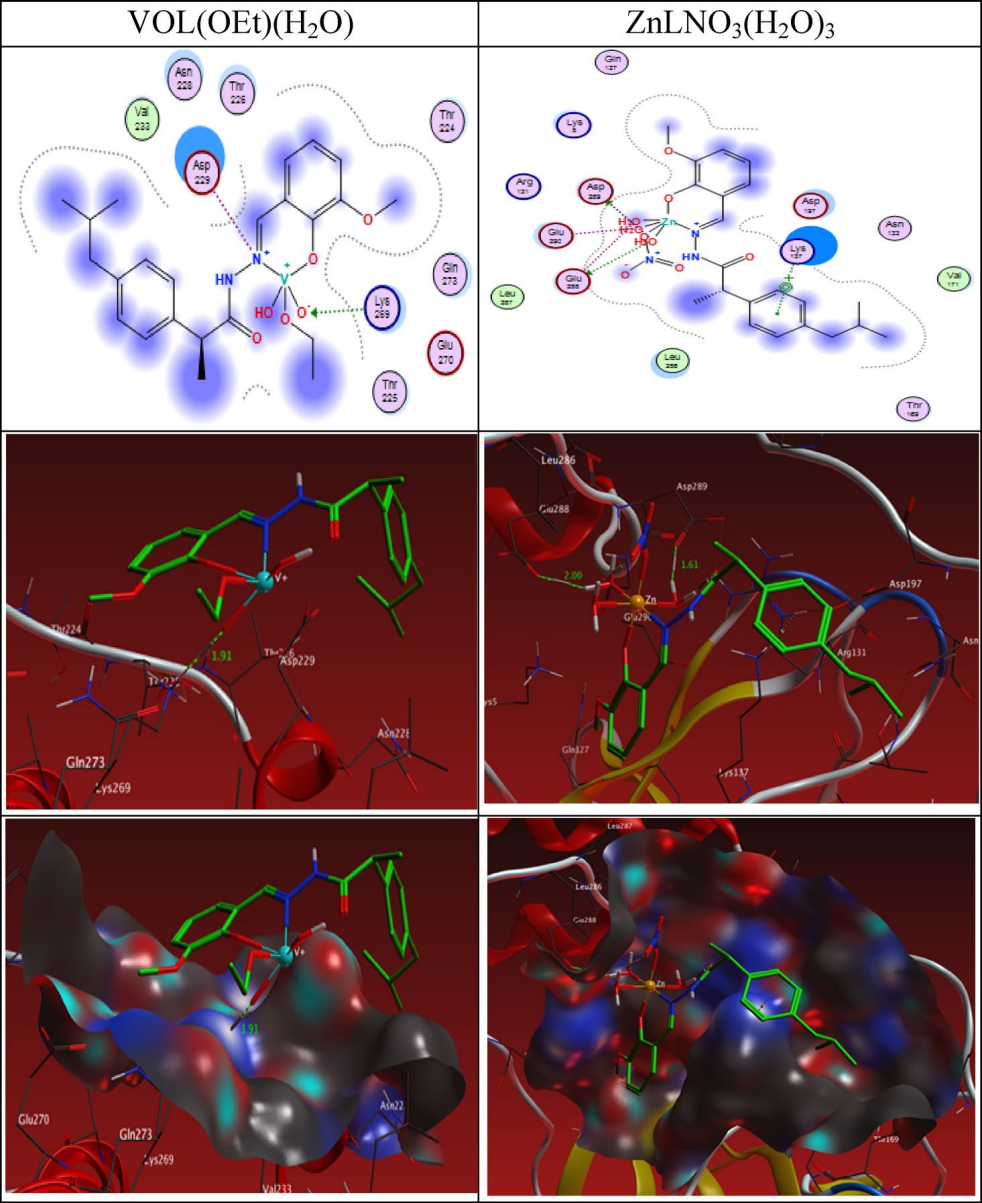
2D and 3D plots of the interaction between VOL(OEt)(H_2_O) and ZnLNO_3_(H_2_O)_3_ with the active site of the receptor of viral protein (PDB ID: 6lu7). Hydrophobic interactions with amino acid residues are shown with dotted curves.

The 2D and 3D plots of the interaction of HL, CuLCl(H_2_O), NiLNO_3_(H_2_O), VOL(OEt)(H_2_O), and ZnLNO_3_(H_2_O)_3_ with the active site of the receptor of COVID-19 main protease viral protein (PDB ID: 6lu7) are shown in Figs. 19 and 20.

#### Cyclooxygenase-2 (PDB:6COX) was obtained from the protein data bank

The binding free energy of ligand and complex with the receptor of active sites of cyclooxygenase-2 (PDB ID: 6COX) are found to be -6.2, -37.2, -32.3, -24.2 and -48.5 kcal/mol for ligand [CuL(H_2_O)*Cl*], [NiL(H_2_O)NO_3_], [VOL(H_2_O)OEt] and [ZnL(H_2_O)_3_NO_3_] complexes; respectively, Table 11. The more negative the binding energy, the stronger the interaction. So, the interactions are in the order of HL ˂ [VOL(H_2_O)OEt] ˂ [NiL(H_2_O)NO_3_] ˂ [CuL(H_2_O)*Cl*] ˂ [ZnL(H_2_O)_3_NO_3_].

**Table 11:** The Docking interaction data calculations of the ligand and its complexes with the active sites of the receptor of cyclooxygenase-2 (PDB ID: 6COX).

	Receptor	Interaction	Distance(Å)*	E (kcal/mol)
HL				
O 24	NH1 AR 44	H-acceptor	2.77 (1.79)	-6.2
[CuL(H_2_O)*Cl*]				
O 2	OD1 ASP 362	H-donor	3.09 (2.35)	-2.0
O 2	OD2 ASP 362	H-donor	2.76 (1.82)	-21.5
O 2	OE1 GLU 364	H-donor	3.30 (2.47)	-0.9
O 2	OD1 ASP 362	Ionic	3.09	-3.9
O 2	OD2 ASP 362	Ionic	2.76	-6.3
O 2	OE1 GLU 364	Ionic	3.30	-2.8
[NiL(H_2_O)NO_3_]				
O 2	O ARG 44	H-donor	2.54 (1.52)	-17.7
C 3	OD1 ASP 125	H-donor	3.21 (2.40)	-0.5
C 10	OD1 ASP 125	H-donor	3.05 (2.13)	-4.3
C 10	OD2 ASP 125	H-donor	3.18 (2.33)	-2.1
O 27	NH1 ARG 44	H-acceptor	2.64 (1.76)	-1.7
O 2	OE2 GLU 46	Ionic	3.07	-4.0
N 12	OD2 ASP 125	Ionic	3.47	-2.0
[VOL(H_2_O)OEt]				
C 10	OE1 GLU 319	H-donor	3.13 (2.27)	-2.1
O 2	NZ LYS 56	H-acceptor	3.09 (2.23)	-1.5
O 29	NZ LYS 56	H-acceptor	2.79 (1.84)	-13.9
O 29	NZ LYS 56	Ionic	2.79	-6.0
6-ring	N GLU 553	pi-H	4.01	-0.7
[ZnL(H_2_O)_3_NO_3_]				
O 2	OE1 GLU 553	H-donor	2.75 (2.04)	-5.3
C 22	SG CYS 59	H-donor	3.81 (2.81)	-0.7
O 59	OD1 ASP 58	H-donor	2.79 (1.85)	-21.6
O 63	NZ LYS 56	H-acceptor	3.17 (2.24)	-3.1
O 2	OE1 GLU 553	Ionic	2.75	-6.4
O 2	OE2 GLU 553	Ionic	2.87	-5.4
O 59	OD1 ASP 58	Ionic	2.79	-6.0

*The lengths of H-bonds are in brackets.

**Fig. 21:**
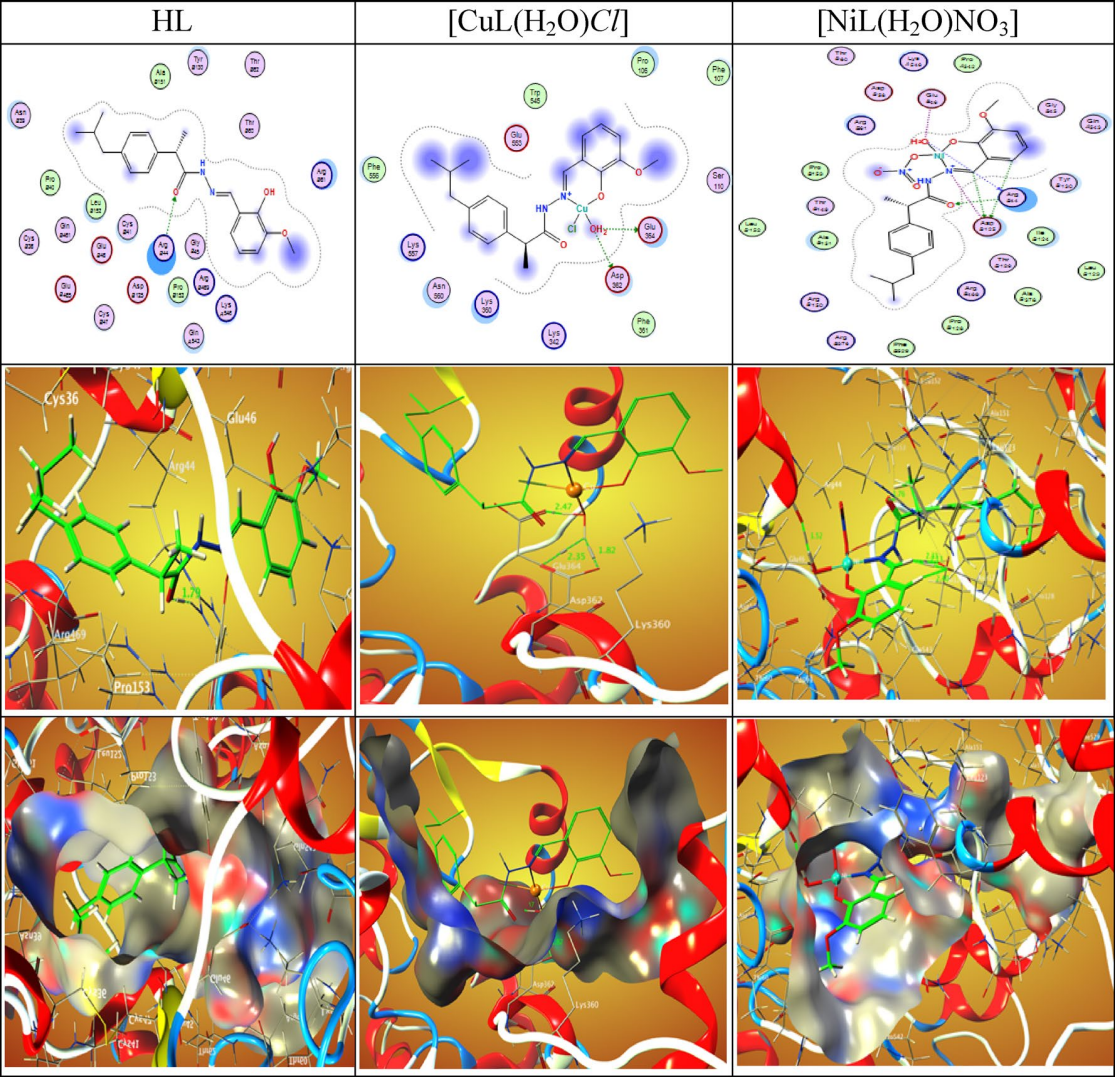
2D and 3D plots of the interaction between the ligand, [CuL(H_2_O)*Cl*] and [NiL(H_2_O)NO_3_] with the active site of cyclooxygenase-2 (PDB ID: 6COX). Hydrophobic interactions with amino acid residues are shown with dotted curves.

**Fig. 22:**
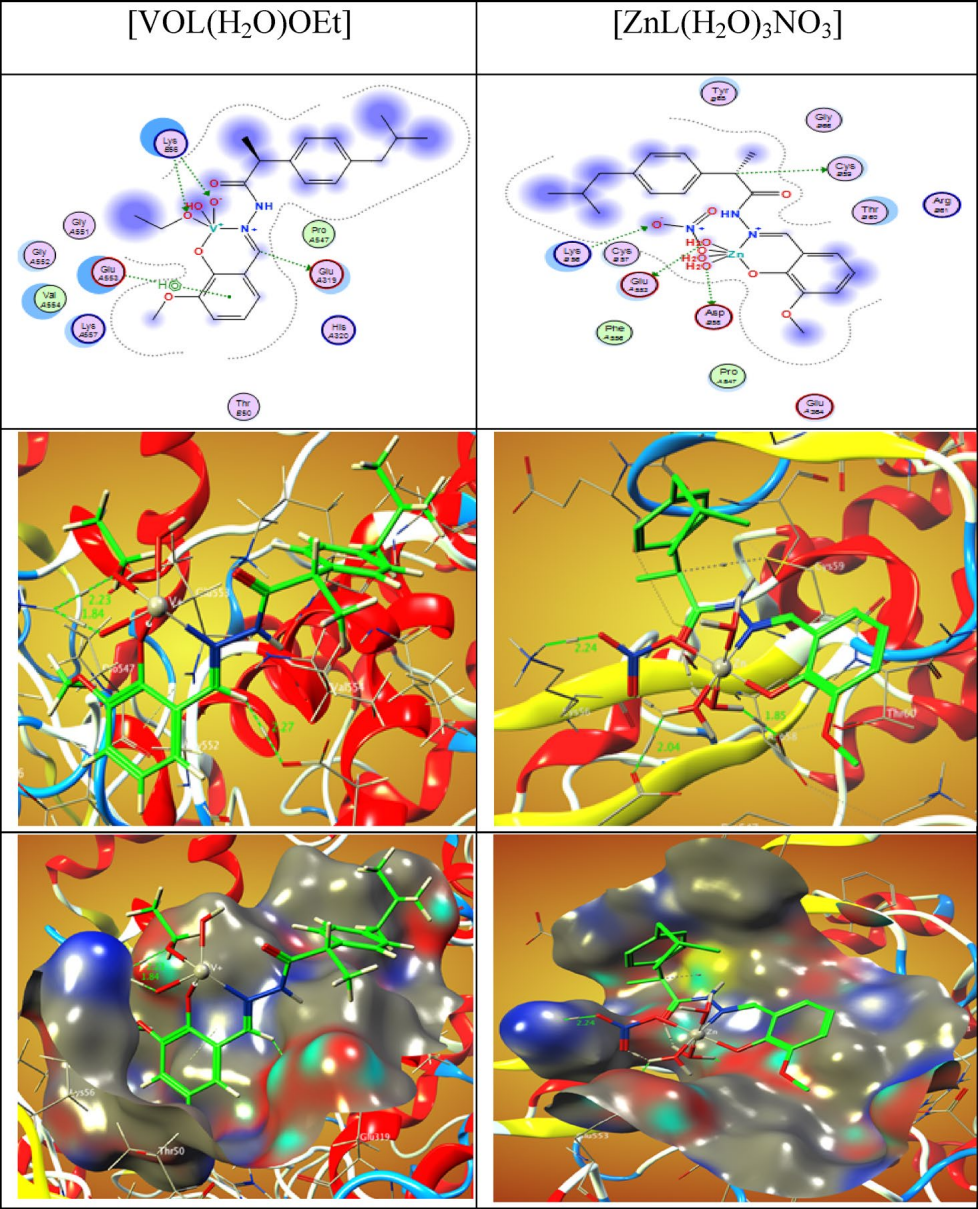
2D and 3D plots of the interaction between [VOL(H_2_O)OEt] and [ZnL(H_2_O)_3_NO_3_] with the active site of the receptor of cyclooxygenase-2 (PDB ID: 6COX). Hydrophobic interactions with amino acid residues are shown with dotted curves.

The 2D and 3D plots of the interaction of the ligand and its complexes with the active site of the receptor of cyclooxygenase-2 (PDB ID: 6COX) are shown in Figs. 21 and 22.

### In silico pharmacokinetic indexes

Clinical development pathways often encounter the abandonment of lead compounds due to suboptimal ADMET (Absorption, Distribution, Metabolism, Excretion, and Toxicity) profiles. Computational modeling allows early-stage drug designers to predict pharmacokinetic failures *before* the commencement of preclinical testing. This virtual screening approach conserves significant resources and accelerates the development timeline by eliminating nonviable candidates during the molecular design phase^48–50^.

The pharmacokinetic profiles of both Schiff base ligands and their corresponding metal complexes were evaluated using the SWISS ADME computational platform. This analysis employed Lipinski’s rule of five criteria to assess the drug-likeness potential, with comprehensive results detailed in Table 12

**Table 12:** Some selected pharmacokinetics data calculated for ligand, [CuL(H_2_O)Cl_2_], [NiL(H_2_O)Cl_2_], [VOL(H_2_O)OEt] and [ZnL(H_2_O)_3_NO_3_] complexes.

ADME FACTOR^a^	Ligand	Cu-complex	Ni-complex	V-complex	Zn-complex
Physicochemical Properties					
Molar Refractivity	107.16	115.74	120.13	107.91	112.86
TPSA	74.41 Å^2^	69.15 Å^2^	124.20 Å^2^	95.45 Å^2^	140.54 Å^2^
Lipophilicity					
Log *P*_o/w_ (iLOGP)	3.57	0.00	0.00	0.00	0.00
Log *P*_o/w_ (XLOGP3)	5.08	4.98	5.21	2.83	2.85
Log *P*_o/w_ (WLOGP)	4.69	4.23	3.73	3.02	2.24
Log *P*_o/w_ (MLOGP)	3.52	2.13	0.73	0.03	-1.09
Log *P*_o/w_ (SILICOS-IT)	5.14	1.34	-1.51	-0.22	-5.34
Consensus Log *P*_o/w_	4.40	2.54	1.63	1.13	-0.27
Water Solubility					
Log *S* (ESOL)	-5.12	-5.74	-5.86	-4.20	-4.54
Class	Moderately soluble	Moderately soluble	Moderately soluble	Moderately soluble	Soluble
Log *S* (Ali)	-6.39	-6.17	-7.57	-4.49	-2.21
Class	Poorly soluble	Poorly soluble	Poorly soluble	Moderately soluble	Moderately soluble
Log *S* (SILICOS-IT)	-5.70	-7.29	-6.26	-4.44	-5.46
Class	Moderately soluble	Poorly soluble	Poorly Soluble	Moderately soluble	Moderately soluble
Pharmacokinetics					
GI absorption	High	High	High	High	Low
BBB permeant	No	Yes	No	No	No
P-gp substrate	No	Yes	Yes	Yes	No
CYP1A2 inhibitor	Yes	No	No	No	No
CYP2C19 inhibitor	No	No	Yes	No	No
CYP2C9 inhibitor	No	No	Yes	No	No
CYP2D6 inhibitor	No	Yes	No	Yes	No
CYP3A4 inhibitor	No	Yes	Yes	Yes	No
Log *K*_p_ (skin permeation)	-4.86 cm/s	-5.63 cm/s	-5.60 cm/s	-6.98 cm/s	-5.82 cm/s
Medicinal Chemistry					
PAINS	1 alert: hzone_phenol_A	0 alert	0 alert: hzone_phenol_A	0 alert	1 alert: hzone_phenol_A
Brenk	2 alerts: imine_1, imine_2	0 alert	2 alerts: nitro_group, oxygen-nitrogen_single_bond	0 alert	2 alerts: imine_1, imine_2
Synthetic accessibility	3.88	5.14	5.08	5.03	3.88

^a^ TPSA:Topological Polar Surface Area), Consensus LogP_o/w_ :Average of all five.

The in silico ADMET (Absorption, Distribution, Metabolism, Elimination, Toxicity) assay is a method used to determine whether a compound possesses acceptable pharmacokinetic and pharmacodynamic properties before administration to the body. The penetration of the blood–brain barrier (BBB), partition coefficient (LogP₀/w), gastrointestinal absorption (GI), topological polar surface area (TPSA), solubility, and skin permeation (Log Kp) of both the ligand and its complex were evaluated using the Swiss ADME server ^51^.

The results indicate that the studied complex demonstrates a high probability of human gastrointestinal absorption (GI) while showing no permeability across the blood–brain barrier, characteristics that are indicative of an ideal drug candidate^52,53^.

## Conclusion

The HL ligand coordinates with the metal ions in a 1:1 molar ratio as an N,O bidentate ligand. The CuL and VOL complexes exhibit octahedral and square-pyramidal geometries, while the ZnL complex is octahedral, and the CuL and NiL complexes have tetrahedral and square planar geometries, respectively.

The Cu(II) complex demonstrated the greatest antibacterial and antifungal efficacy. Cytotoxicity tests on HCT116, HepG2, and MCF7 cell lines revealed that CuL was the most effective. Such findings lead to conclude that the Schiff base complexes of ibuprofen and o-vanillin have significant potential as effective antimicrobial agents for medical applications.

## Supplementary Information

Below is the link to the electronic supplementary material.

Supplementary Material 1

## Data Availability

All data generated or analysed during this study are included in this published article [and its supplementary information files].

## References

[1] Fries, J. F., Williams, C. A., Bloch, D. A. & Michel, B. A. Nonsteroidal anti-inflammatory drug-associated gastropathy: Incidence and risk factor models. *Am. J. Med.* **91**(3), 213–222. 10.1016/0002-9343(91)90118-H (1991). 1892140 10.1016/0002-9343(91)90118-h

[2] Basha, M. T., Alghanmi, R. M., Shehata, M. R. & Abdel-Rahman, L. H. Synthesis, structural characterization, DFT calculations, biological investigation, molecular docking and DNA binding of Co(II), Ni(II) and Cu(II) nanosized Schiff base complexes bearing pyrimidine moiety. *J. Mol. Struct.* **1183**, 298–312. 10.1016/j.molstruc.2019.02.001 (2019).

[3] Zettl, M. et al. Characterization of a novel drying technology for continuous processing of cohesive materials: An Ibuprofen case study. *Org. Process Res. Dev.* **25**(4), 769–780. 10.1021/acs.oprd.0c00413 (2021).

[4] Ali Alderawy, M. Q., Raheem Alrubaie, L. A. & Sheri, F. H. Synthesis, characterization of ibuprofen n-acyl-1,3,4 oxadiazole derivatives and anticancer activity against MCF-7 cell line. *Syst. Rev. Pharm.* **11**(4), 681–689. 10.31838/srp.2020.4.100 (2020).

[5] Manzano, C. M. et al. Pt(II) and Pd(II) complexes with ibuprofen hydrazide: Characterization, theoretical calculations, antibacterial and antitumor assays and studies of interaction with CT-DNA. *J. Mol. Struct.* **1154**(Ii), 469–479. 10.1016/j.molstruc.2017.10.072 (2018).

[6] Abdel-Mawgoud, A. M., Ismael, M. & Abdou, A. Synthesis, characterization, antimicrobial evaluation and DFT calculations of Fe(III), Ni(II) and Cu(II) complexes of tridentate ONO donor ligand. *J. Pharm. Appl. Chem.* **3**(3), 259–266. 10.18576/jpac/030309 (2017).

[7] Connors, K. A. The stability of cyclodextrin complexes in solution. *Chem. Rev.* **97**(5), 1325–1357. 10.1021/cr960371r (1997). 11851454 10.1021/cr960371r

[8] Abu-Dief, A. M. et al. Synthesis and characterization of new Cr(III), Fe(III) and Cu(II) complexes incorporating multi-substituted aryl imidazole ligand: Structural, DFT, DNA binding, and biological implications. *Spectrochim. Acta Part A Mol. Biomol. Spectrosc.* **228**, 117700. 10.1016/j.saa.2019.117700 (2020). 10.1016/j.saa.2019.117700 31748163

[9] Abdel-rahman, L. H., Abu-dief, A. M. & Abdel-mawgoud, A. A. H. Development, structural investigation, DNA binding, antimicrobial screening and anticancer activities of two novel quari-dentate VO (II) and Mn (II) mononuclear complexes. *J. King Saud Univ. - Sci.* **31**(1), 52–60. 10.1016/j.jksus.2017.05.011 (2019).

[10] Ellis, J. K., Lucero, M. J. & Scuseria, G. E. The indirect to direct band gap transition in multilayered MoS 2 as predicted by screened hybrid density functional theory. *Appl. Phys. Lett.*10.1063/1.3672219 (2011).

[11] Hay, P. J. & Wadt, W. R. Ab initio effective core potentials for molecular calculations. Potentials for K to Au including the outermost core orbitale. *J. Chem. Phys.* **82**(1), 299–310. 10.1063/1.448975 (1985).

[12] Abu-dief, A. M., Nassar, I. F. & Elsayed, W. H. Magnetic NiFe_2_O_4_ nanoparticles: Efficient, heterogeneous and reusable catalyst for synthesis of acetylferrocene chalcones and their anti-tumour activity. *Appl. Organomet. Chem.*10.1002/aoc.3521 (2016).

[13] Elkanzi, N. A. A., Ali, A. M., Hrichi, H. & Abdou, A. New mononuclear Fe(III), Co(II), Ni(II), Cu(II), and Zn(II) complexes incorporating 4-{[(2 hydroxyphenyl)imino]methyl}phenyl-4-methylbenzenesulfonate (HL): Synthesis, characterization, theoretical, anti-inflammatory, and molecular docking investigation. *Appl. Organomet. Chem.* **36**(5), 1–16. 10.1002/aoc.6665 (2022).

[14] Abdel-Rahman, L. H., El-Khatib, R. M., Nassr, L. A. E. & Abu-Dief, A. M. Synthesis, physicochemical studies, embryos toxicity and DNA interaction of some new Iron(II) Schiff base amino acid complexes. *J. Mol. Struct.* **1040**, 9–18. 10.1016/j.molstruc.2013.02.023 (2013).

[15] Abdel-rahman, L. H., Ismail, N. M., Ismael, M., Abu-dief, A. M. & Ahmed, E. A. Synthesis, characterization, DFT calculations and biological studies of Mn (II), Fe (II), Co (II) and Cd (II) complexes based on a tetradentate ONNO donor Schiff base ligand. *J. Mol. Struct.* **1134**, 851–862. 10.1016/j.molstruc.2017.01.036 (2017).

[16] Vanparia, S. F. et al. Synthesis, characterization and antimicrobial study of novel 4-{[(8-Hydroxyquinolin-5-yl) methyl] amino} benze- nesulfonamide and its oxinates. *Acta Chim. Slov.* 660–667 (2010). 24061814

[17] Konstantinovi, S. S., Radovanovi, B. C. & Caki, I. Synthesis and characterization of Co (II), Ni (II), Cu (II) and Zn (II) complexes with 3-salicylidenehydrazono-2-indolinone. *JSCS–***68**, 641–647 (2003).

[18] Sun, J. et al. Visible-light-induced iminyl radical formation via electron-donor–acceptor complexes: A photocatalyst-free approach to phenanthridines and quinolines†. *Org. Chem.*10.1039/c7qo00992e (2018).

[19] Gaber, M., Fayed, T. A., El-Nahass, M. N., Diab, H. A. & El-Gamil, M. M. Synthesis, spectroscopic characterization and biological evaluation of a novel chemosensor with different metal ions. *Appl. Organomet. Chem.* **33**(11), 1–16. 10.1002/aoc.5133 (2019).

[20] Sengupta, S. K., Pandey, O. P., & Srivastava, B. K. Synthesis, structural and biochemical aspects of titanocene and zirconocene chelates of acetylferrocenyl thiosemicarbazones. *Transit. Met. Chem.* **353** (1998).

[21] Pettersen, E. F. et al. UCSF Chimera—A visualization system for exploratory research and analysis. *J. Comput. Chem.* **25**(13), 1605–1612. 10.1002/jcc.20084 (2004). 15264254 10.1002/jcc.20084

[22] Abdel-Rahman, L. H., Basha, M. T., Al-Farhan, B. S., Shehata, M. R. & Abdalla, E. M. Synthesis, characterization, potential antimicrobial, antioxidant, anticancer, DNA binding, and molecular docking activities and DFT on novel Co(II), Ni(II), VO(II), Cr(III), and La(III) Schiff base complexes. *Appl. Organomet. Chem.* **36**(1), 1–29. 10.1002/aoc.6484 (2022).

[23] Gupta, S. K. et al. An unusual hydroxy-substituted mononuclear nickel ( II ) complex with a tetradentate Schiff base: Synthesis, spectroscopy, electrochemistry, crystallography, DNA binding, and theoretical investigation. *Polyhedron* **89**, 219–231. 10.1016/j.poly.2015.01.017 (2015).

[24] Zaky, R. R., Ibrahim, K. M. & El-nadar, H. M. A. Bivalent transition metal complexes of ( E ) -3- (2-benzylidenehydrazinyl ) -3-oxo- N - ( p -tolyl ) propanamide: Spectroscopic, computational, biological activity studies. *Spectrochim. ACTA PART A Mol. Biomol. Spectrosc.* **150**, 40–53. 10.1016/j.saa.2015.04.107 (2015). 10.1016/j.saa.2015.04.107 26023055

[25] Abdel-rahman, L. H., El-khatib, R. M., Nassr, L. A. E. & Abu-dief, A. M. DNA binding ability mode, spectroscopic studies, hydrophobicity, and in vitro antibacterial evaluation of some new Fe (II) complexes bearing ONO donors amino acid Schiff bases. *Arab. J. Chem.* **10**, S1835–S1846. 10.1016/j.arabjc.2013.07.010 (2017).

[26] Srivastva, A. N., Singh, N. P. & Shriwastaw, C. K. In vitro antibacterial and antifungal activities of binuclear transition metal complexes of ONNO Schiff base and 5-methyl-2,6-pyrimidine-dione and their spectroscopic validation. *Arab. J. Chem.* **9**(1), 48–61. 10.1016/j.arabjc.2014.10.004 (2016).

[27] Anitha, C., Sheela, C. D., Tharmaraj, P. & Sumathi, S. Spectroscopic studies and biological evaluation of some transition metal complexes of azo Schiff-base ligand derived from (1-phenyl-2,3-dimethyl-4- aminopyrazol-5-one) and 5-((4-chlorophenyl)diazenyl)-2-hydroxybenzaldehyde. *Spectrochim. Acta Part A Mol. Biomol. Spectrosc.* **96**, 493–500. 10.1016/j.saa.2012.05.053 (2012). 10.1016/j.saa.2012.05.053 22728967

[28] Loginova, N. V. et al. Redox-active antifungal cobalt(II) and copper(II) complexes with sterically hindered o-aminophenol derivatives. *Polyhedron* **27**(3), 985–991. 10.1016/j.poly.2007.11.028 (2008).

[29] Abdel-rahman, L. H., Abu-dief, A. M., Moustafa, H. & Abdel-mawgoud, A. A. H. Design and nonlinear optical properties (NLO) using DFT approach of new Cr (III), VO (II), and Ni (II) chelates incorporating tri-dentate imine ligand for DNA interaction, antimicrobial, anticancer activities and molecular docking studies. *Arab. J. Chem.* **13**(1), 649–670. 10.1016/j.arabjc.2017.07.007 (2020).

[30] Rosnizam, A. N. et al. Palladium(II) complexes bearing N, O-bidentate Schiff base ligands: Experimental, in-silico, antibacterial, and catalytic properties. *J. Mol. Struct.*10.1016/j.molstruc.2022.132821 (2022).

[31] Abdel-Rahman, L. H., Ismail, N. M., Ismael, M., Abu-Dief, A. M. & Ahmed, E. A. H. Synthesis, characterization, DFT calculations and biological studies of Mn(II), Fe(II), Co(II) and Cd(II) complexes based on a tetradentate ONNO donor Schiff base ligand. *J. Mol. Struct.* **1134**, 851–862. 10.1016/j.molstruc.2017.01.036 (2017).

[32] Williams, L. A. D. et al. The in vitro anti-denaturation effects induced by natural products and non-steroidal compounds in heat treated (Immunogenic) bovine serum albumin is proposed as a screening assay for the detection of anti-inflammatory compounds, without the use of animals. *West Indian Med. J.* **57**(4), 327–331 (2008). 19566010

[33] Abdel-rahman, L. H., El-khatib, R. M., Nassr, L. A. E. & Abu-dief, A. M. Synthesis, physicochemical studies, embryos toxicity and DNA interaction of some new Iron (II) Schiff base amino acid complexes. *J. Mol. Struct.* **1040**, 9–18. 10.1016/j.molstruc.2013.02.023 (2013).

[34] Abdel-Rahman, L. H. et al. Synthesis, theoretical investigations, biocidal screening, DNA binding, in vitro cytotoxicity and molecular docking of novel Cu (II), Pd (II) and Ag (I) complexes of chlorobenzylidene Schiff base: Promising antibiotic and anticancer agents. *Appl. Organomet. Chem.* **32**(12), 1–21. 10.1002/aoc.4527 (2018).

[35] El-Shafiy, H. F. & Shebl, M. Binuclear oxovanadium(IV), cerium(III) and dioxouranium(VI) nano complexes of a bis(bidentate) ligand: Synthesis, spectroscopic, thermal, DFT calculations and biological studies. *J. Mol. Struct.* **1194**, 187–203. 10.1016/j.molstruc.2019.05.063 (2019).

[36] El-Shafiy, H. F. & Shebl, M. Oxovanadium(IV), cerium(III), thorium(IV) and dioxouranium(VI) complexes of 1-ethyl-4-hydroxy-3-(nitroacetyl)quinolin-2(1H)-one: Synthesis, spectral, thermal, fluorescence, DFT calculations, antimicrobial and antitumor studies. *J. Mol. Struct.* **1156**, 403–417. 10.1016/j.molstruc.2017.11.081 (2018).

[37] Shebl, M. Synthesis, spectroscopic characterization and antimicrobial activity of binuclear metal complexes of a new asymmetrical Schiff base ligand: DNA binding affinity of copper(II) complexes. *Spectrochim. Acta Part A Mol. Biomol. Spectrosc.* **117**, 127–137. 10.1016/j.saa.2013.07.107 (2014). 10.1016/j.saa.2013.07.107 23988527

[38] Mahmoud, W. H., Deghadi, R. G. & Mohamed, G. G. Novel Schiff base ligand and its metal complexes with some transition elements. Synthesis, spectroscopic, thermal analysis, antimicrobial and in vitro anticancer activity. *Appl. Organomet. Chem.* **30**(4), 221–230. 10.1002/aoc.3420 (2016).

[39] Devi, J., Kumar, S., Kumar, B., Asija, S. & Kumar, A. *Synthesis, structural analysis, in vitro antioxidant, antimicrobial activity and molecular docking studies of transition metal complexes derived from Schiff base ligands of 4-(benzyloxy)-2-hydroxybenzaldehyde*, vol. 48, no. 4 (Springer, 2022). 10.1007/s11164-021-04644-y.

[40] Muthukumarasamy, N., Jayakumar, S., Kannan, M. D., Balasundaraprabhu, R. & Ramanathaswamy, P. Structural and optical properties of hot wall deposited CdSe 0.15Te0.85 thin films. *J. Cryst. Growth* **263**(1–4), 308–315. 10.1016/j.jcrysgro.2003.11.081 (2004).

[41] Fahim, A. M., Magar, H. S., Nasar, E., Abdelrazek, F. M. & Aboelnaga, A. Synthesis of Cu-porphyrazines by annulated diazepine rings with electrochemical, conductance activities and computational studies. *J. Inorg. Organomet. Polym. Mater.* **32**(1), 240–266. 10.1007/s10904-021-02122-x (2022).

[42] Anacona, J. R., Rodriguez, J. L. & Camus, J. Synthesis, characterization and antibacterial activity of a Schiff base derived from cephalexin and sulphathiazole and its transition metal complexes. *Spectrochim. Acta Part A Mol. Biomol. Spectrosc.* **129**, 96–102. 10.1016/j.saa.2014.03.019 (2014). 10.1016/j.saa.2014.03.019 24727167

[43] Garribba, E. & Micera, G. The determination of the geometry of Cu (II) complexes: An EPR spectroscopy experiment. *J. Chem. Educ.* **83**(8), 1229 (2006).

[44] Wang, Y. T. et al. A new 2-D cobalt coordination polymer with the flexible 2-(1H-imidazole-1-yl)acetate: Synthesis, structure, and characterization. *J. Coord. Chem.* **63**(9), 1504–1513. 10.1080/00958972.2010.481717 (2010).

[45] Gurusamy, S., Krishnaveni, K., Sankarganesh, M., Nandini Asha, R. & Mathavan, A. Synthesis, characterization, DNA interaction, BSA/HSA binding activities of VO(IV), Cu(II) and Zn(II) Schiff base complexes and its molecular docking with biomolecules. *J. Mol. Liq.* **345**, 117045. 10.1016/j.molliq.2021.117045 (2022).

[46] Nishat, N., Zulfequar, M., Asma, & Hasnain, S. Synthesis, spectral, and antibacterial screening studies of chelating polymers of bisphenol-A-formaldehyde resin bearing barbituric acid. *J. Coord. Chem.* **63**(7), 1273–1281. 10.1080/00958971003728296 (2010).

[47] Nejo, A. A., Kolawole, G. A. & Nejo, A. O. Synthesis, characterization, antibacterial, and thermal studies of unsymmetrical Schiff-base complexes of cobalt(II). *J. Coord. Chem.* **63**(24), 4398–4410. 10.1080/00958972.2010.532871 (2010).

[48] Abdel-Rahman, L. H., Abu-Dief, A. M. & Hassan Abdel-Mawgoud, A. A. Development, structural investigation, DNA binding, antimicrobial screening and anticancer activities of two novel quari-dentate VO(II) and Mn (II) mononuclear complexes. *J. King Saud Univ. Sci.* **31**(1), 52–60. 10.1016/j.jksus.2017.05.011 (2019).

[49] Mahmoud, W. H., Deghadi, R. G. & Mohamed, G. G. Metal complexes of novel Schiff base derived from iron sandwiched organometallic and 4-nitro-1,2-phenylenediamine: Synthesis, characterization, DFT studies, antimicrobial activities and molecular docking. *Appl. Organomet. Chem.* **32**(4), 1–22. 10.1002/aoc.4289 (2018).

[50] Ahmed, Y. M. & Mohamed, G. G. Synthesis, spectral characterization, antimicrobial evaluation and molecular docking studies on new metal complexes of novel Schiff base derived from 4,6-dihydroxy-1,3-phenylenediethanone. *J. Mol. Struct.*10.1016/j.molstruc.2022.132496 (2022).35698532

[51] Panchal, P. K., Pansuriya, P. B. & Patel, M. N. In-vitro biological evaluation of some ONS and NS donor Schiff’s bases and their metal complexes. *J. Enzyme Inhib. Med. Chem.* **21**(4), 453–458. 10.1080/14756360600628551 (2006). 17059180 10.1080/14756360600628551

[52] Manna, S. C., Mistri, S. & Jana, A. D. A rare supramolecular assembly involving ion pairs of coordination complexes with a host-guest relationship: Synthesis, crystal structure, photoluminescence and thermal study. *CrystEngComm* **14**(21), 7415–7422. 10.1039/c2ce25916h (2012).

[53] Hashem, H. E., Mohamed, E. A., Farag, A. A., Negm, N. A. & Azmy, E. A. M. New heterocyclic Schiff base-metal complex: Synthesis, characterization, density functional theory study, and antimicrobial evaluation. *Appl. Organomet. Chem.* **35**(9), 1–14. 10.1002/aoc.6322 (2021).

