# Supplementary material for: Synthesis and biological evaluation of ibuprofen/o-vanillin Schiff base complexes with anti-inflammatory, anti-proliferative and anti-SARS-COV-19 activities
Source: Sci Rep. 2026 Mar 7;16:8501. doi: 10.1038/s41598-026-38270-8 (PMC12972119; doi:10.1038/s41598-026-38270-8)
Supplement: Supplementary file 1 — Supplementary Material 1 [file 41598_2026_38270_MOESM1_ESM.docx]

**Supplementary data**

**Synthesis and biological evaluation of ibuprofen/o-vanillin Schiff base complexes with anti-inflammatory, anti-proliferative and anti-SARS-COV-2 activities**

Laila H. Abdel-Rahman ^a*^, Doaa Abou El-ezz ^b^, Abdel-Mawgoud M. Abdel-Mawgoud ^a^, Mohamed R. Shehata ^c^, Mahmoud Abd El Aleem Ali Ali El-Remaily^a^, Mohamed Abdel-Hameed ^a,^ and Shaaban K. Mohamed ^d*^

^a^ Chemistry Department, Faculty of Science, Sohag University, 82534 Sohag, Egypt

^b^ Pharmacology and toxicology department, Faculty of Pharmacy, October University for Modern Sciences & Arts, Giza, Egypt

^c^ Chemistry Department, Faculty of Science, Cairo University, Giza, Egypt

^d^ Chemistry and Environmental Division, Manchester Metropolitan University, Manchester M1 6GD, England

*Corresponding authors: E-mail:

[laila.abdelrahman@science.sohag.edu.eg](mailto:laila.abdelrahman@science.sohag.edu.eg) and shaabankamel@yahoo.com

**1. Thermogravimetric analysis (TGA) and Thermo-kinetic parameters**

$\ln\left[ \frac{ln (\frac{W_{\infty}}{W_{\infty}-W})}{T^{2}} \right] = ln \left[ \frac{AR}{\emptyset E^{*}}(1-\frac{2RT}{E^{*}}) \right] - \frac{E^{*}}{2.303 RT}$ **(1)**

In **equation 1**, the left section figure against (1/T) to produce a straight-line relationship. In contrast, (1 − 2RT) ≈ 1

$\Delta H^{*}= E* - RT$ **(2)**

$\Delta S^{*}= 2.303RLog ( \frac{Ah}{K_{B}T} )$ **(3)**

$\Delta G^{*}$= $\Delta H^{*}$-T $\Delta S^{*}$ **(4)**

In which Boltzmann's constant K_B_ and Planck's constant h play integral roles.

**2. ^1^H NMR spectra of HL Ligand and its ZnL Complex**

| 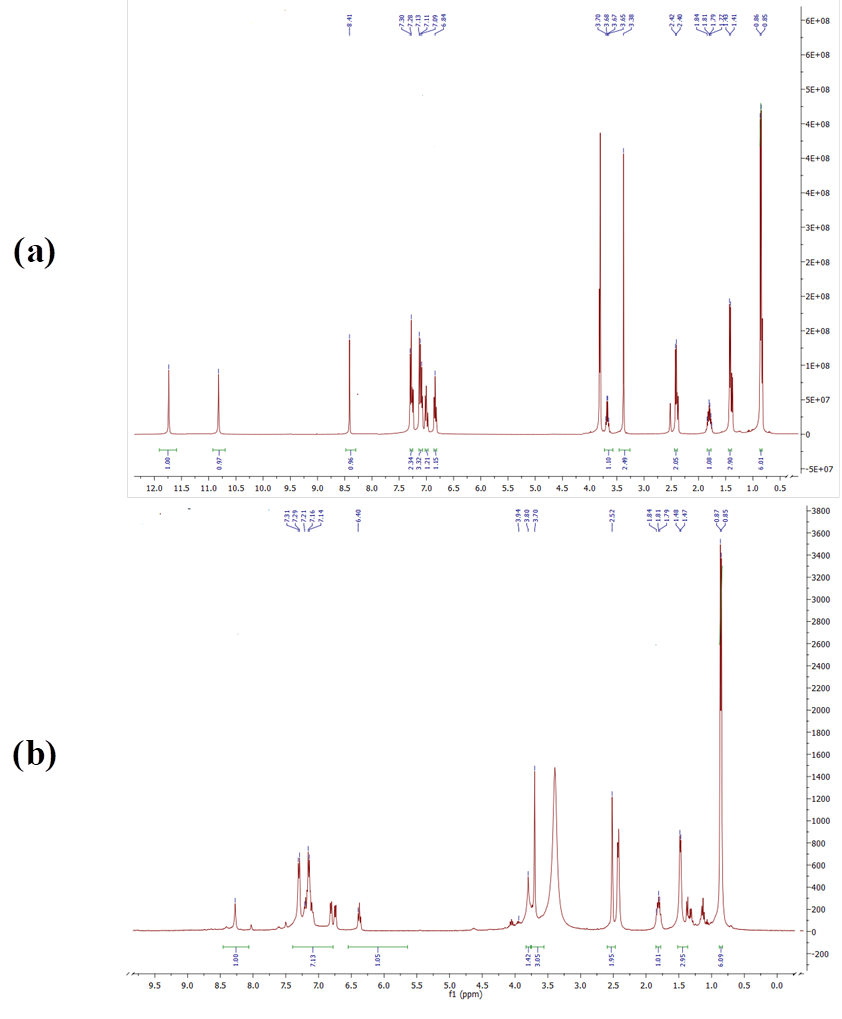 |
| --- |
| 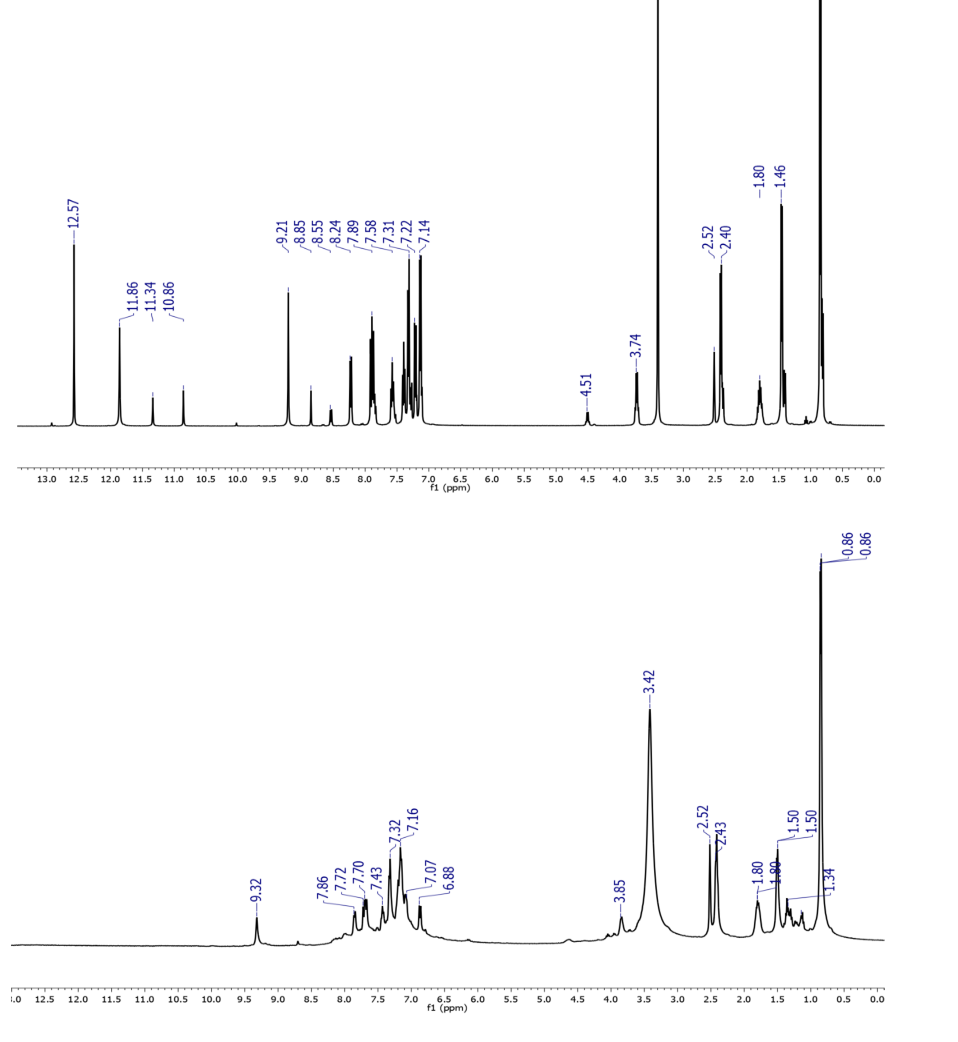 |

**Figure S1:** The ^1^H-NMR spectra of the HL ligand (a) and its ZnL and NiL complexes (b).

**3. FT-IR Spectral Studies**

**Table S1:** Characteristic IR bands (cm^-1^) of the synthesized ligand and their complexes

| **Compound formula** | **IR cm^-1^** | | | | | | |
| --- | --- | --- | --- | --- | --- | --- | --- |
|  | **υ _(OH)/H2O broad_** | **υ_Ph_ _(-N-H)_** | **υ_St_ _(-C= O)_** | **υ_St_ _(-CH= N)_** | **υ _(OH)/H2O_** | **υ_(M-N)_** | **υ_(M-O)_** |
| **HL** |  | **3268** | **1698** | **1612** | **-** | **-** | **-** |
| **VOL** | **3745** | **2953** | **1690** | **1602** | **992** | **422** | **530** |
| **NiL** | **3411** | **2952** | **1660** | **1600** | **932** | **445** | **542** |
| **CuL** | **3544** | **3270** | **1735** | **1604** | **970** | **455** | **513** |
| **ZnL** | **3429** | **3202** | **1708** | **1609** | **966** | **448** | **549** |


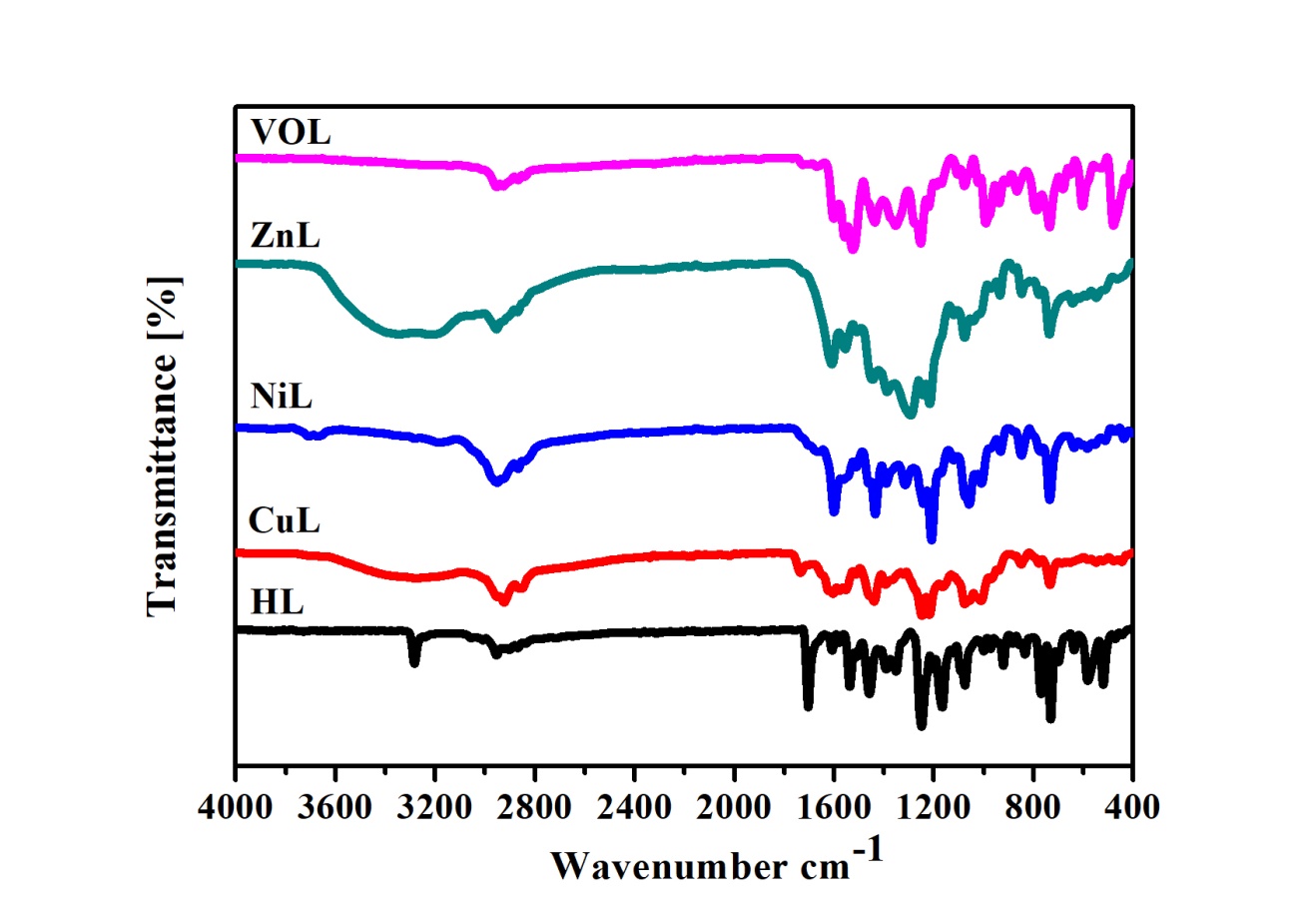


**Figure S2:** FT- IR of the ligand HL and its Cu(II), Ni(II), Zn(II), and VO(II) complexes.

**4**. **Electronic Spectra**

7

**Figure** **S3:** Electronic spectra of HL and its metal complexes in DMF at 298K

**4.** **Mass Spectra**

**
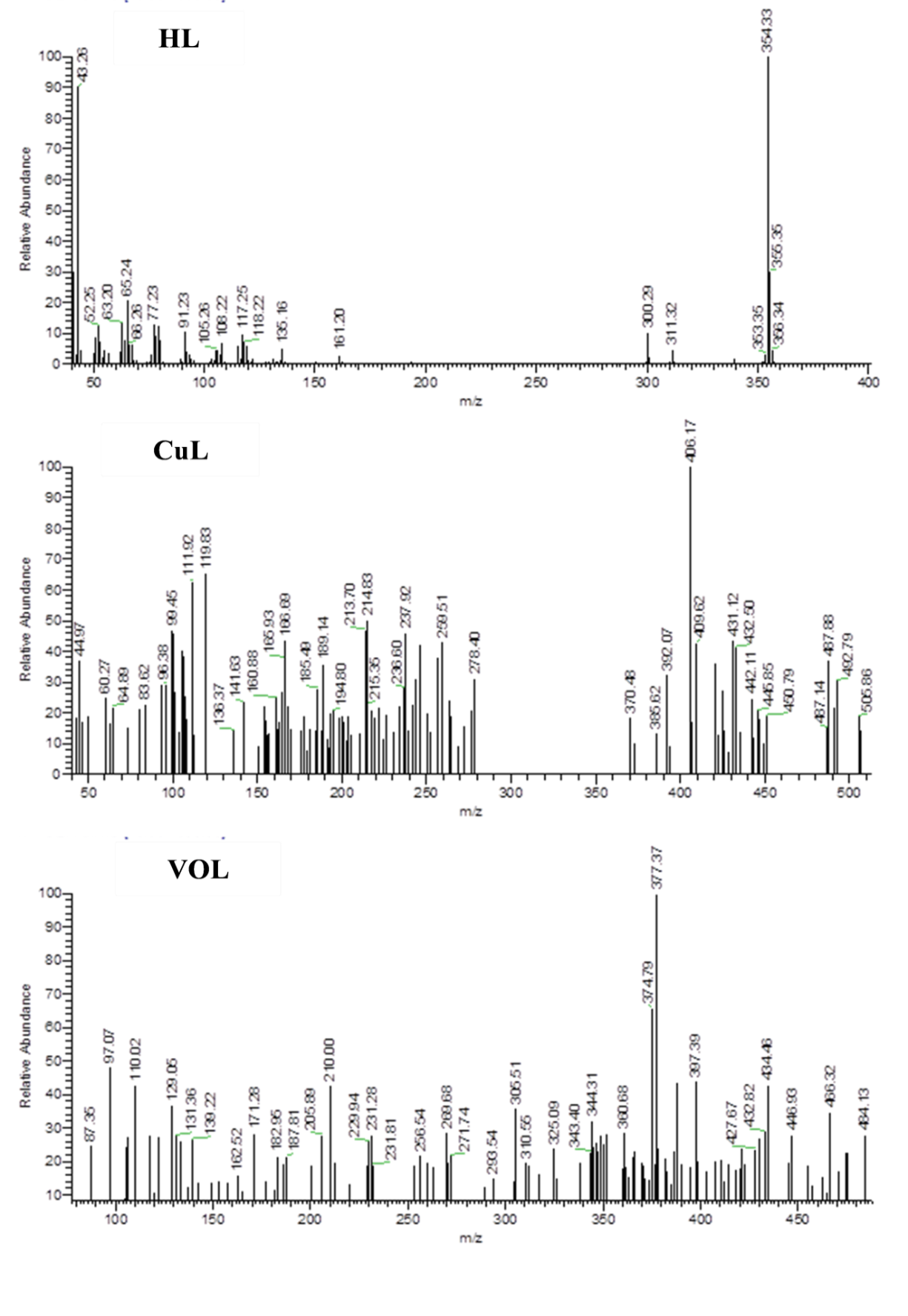
**

**Figure S4:** The mass spectra of ligand and its CuL and VOL Complexes.

**5**. **Complexes stoichiometry**

**Figure S5:** Complex stoichiometry by job method for the new complexes.

**6. Thermogravimetric analysis (TGA) and Thermo-kinetic parameters**

|  |
| --- |
| 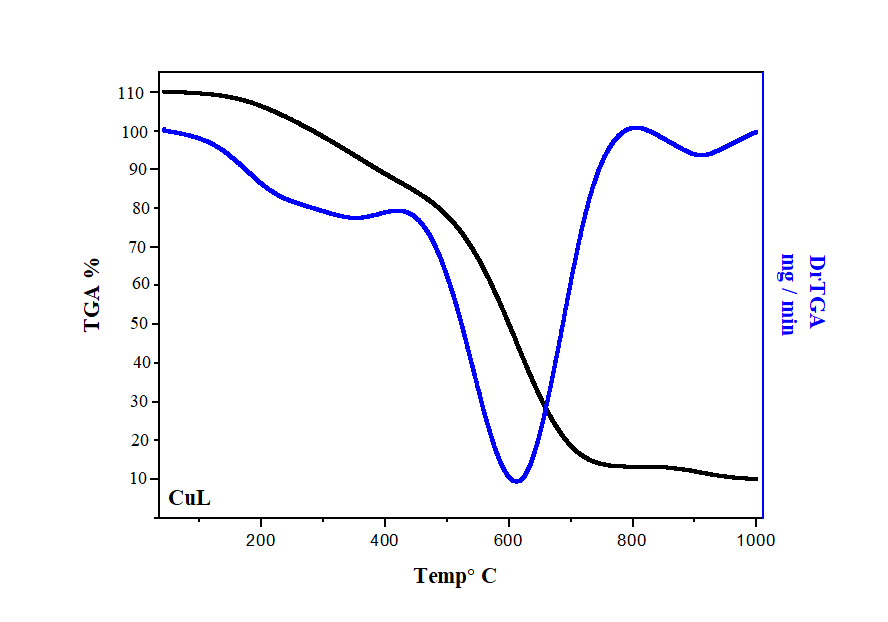 |
|  |
|  |
|   **Figure S6:** TGA and Dr TGA of the Cul, NiL, ZnL and VOL complexes in temperature range 25 – 1000 °C under nitrogen with heating rate of 5 °C per minute.  **7. PXRD study** |
|  |
|  |

**Figure S7:**  PXRD patterns of the VOL and ZnL complexes in 2θ range 0 – 80 °.

**Table 2:** Dislocation density and micro strain values of CuL complex.

| **No** | **2Ө (*)** | **Β (*)** | **D (nm)** | **δ**$\boldsymbol{\times}$ **10^-3^ (nm^-2^)** | **ε** $\boldsymbol{\times}$ **10^-3^** |
| --- | --- | --- | --- | --- | --- |
| 1 | 5.82991 | 2.71214 | 2.932954 | 0.000116 | 0.00023 |
| 2 | 10.98495 | 3.9293 | 2.031133 | 0.000242 | 0.00018 |
| 3 | 12.6311 | 5.711 | 1.399544 | 0.000511 | 0.00023 |
| 4 | 12.82604 | 30.92106 | 0.258539 | 0.014961 | 0.0012 |
| 5 | 13.93069 | 2.33541 | 3.42696 | 8.51E-05 | 8.3E-05 |
| 6 | 15.20862 | 3.49876E-4 | 22907.47 | 1.91E-12 | 1.1E-08 |
| 7 | 16.13999 | 2.23866 | 3.584173 | 7.78E-05 | 6.9E-05 |
| 8 | 17.61286 | 0.40043 | 20.07607 | 2.48E-06 | 1.1E-05 |
| 9 | 19.08573 | 0.21867 | 36.83983 | 7.37E-07 | 5.7E-06 |
| 10 | 20.0171 | 4.13898 | 1.949044 | 0.000263 | 0.0001 |
| 11 | 22.59462 | 1.78391 | 4.541294 | 4.85E-05 | 3.9E-05 |
| 12 | 24.24077 | 14.2984 | 0.568274 | 0.003097 | 0.00029 |
| 13 | 26.64501 | 2.99859 | 2.722611 | 0.000135 | 5.5E-05 |
| 14 | 28.11788 | 1.26334 | 6.482497 | 2.38E-05 | 2.2E-05 |
| 15 | 28.68103 | 0.33557 | 24.4354 | 1.67E-06 | 5.7E-06 |
| 16 | 30.1539 | 4.62238 | 1.779928 | 0.000316 | 7.5E-05 |
| 17 | 31.62677 | 11.64054 | 0.709312 | 0.001988 | 0.00018 |
| 18 | 33.46785 | 4.58658 | 1.808666 | 0.000306 | 6.7E-05 |
| 19 | 35.67715 | 0.82574 | 10.10671 | 9.79E-06 | 1.1E-05 |
| 20 | 9.8803 | 0.12596 | 63.3051 | 2.5E-07 | 6.4E-06 |
| 21 | 45.61902 | 23.46909 | 0.367217 | 0.007416 | 0.00024 |
| 22 | 39.72754 | 10.43141 | 0.809751 | 0.001525 | 0.00013 |

**8. Antifungal activity**

**
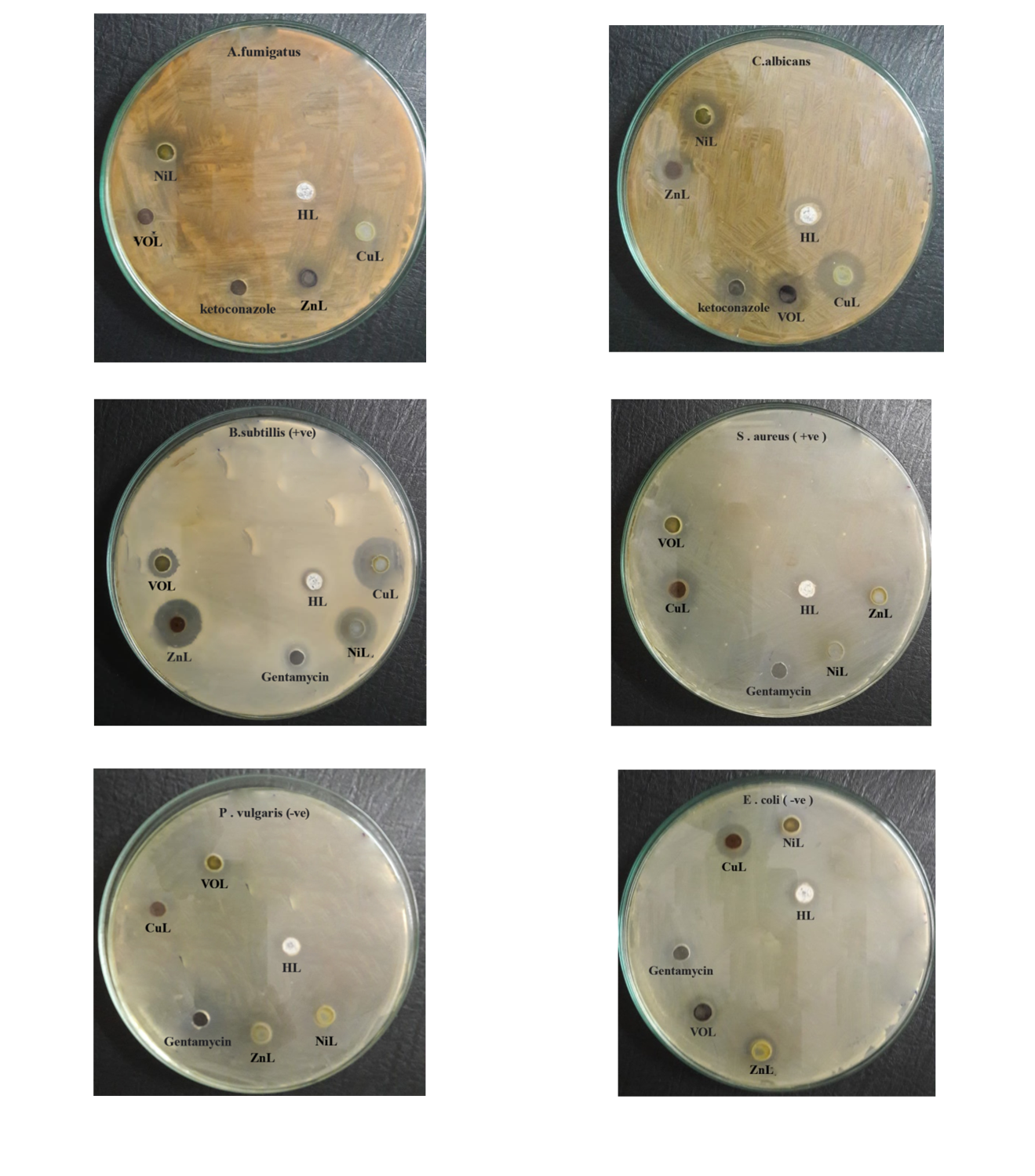
**

**Figure S8**: Antibacterial and antifungal screening data of ibuprofen Schiff base (HL), mononuclear metal complexes ( CuL, NiL, ZnL and VOL ) and standard drug. Agar well diffusion technique was used to evaluate the antibacterial activity of the synthesized compounds.

**9. Gel electrophoresis**


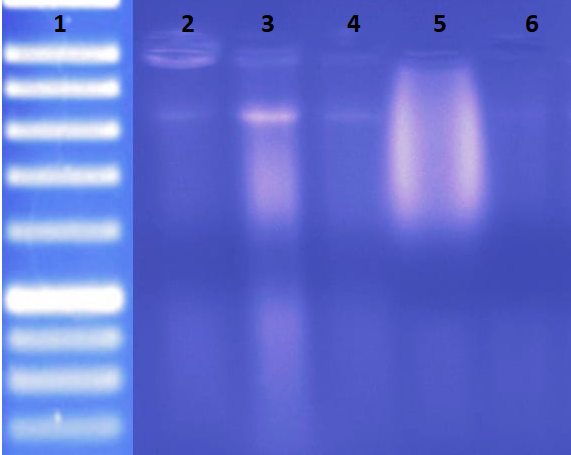


**Figure** **S9**: Gel electrophoresis results for CT-DNA in the presence of the new metal complexes. Lane 1: DNA ladder; lane 2: ZnL complex + CT‐DNA; lane 3: CuL complex + CT‐DNA; lane 4: HL ligand + CT‐DNA; lane 5: NiL complex+ CT‐DNA; lane 6: VOL complex+ CT‐DNA.

**10. Antimicrobial activity**

**Table S3**: Antibacterial and Antifungal screening data of Schiff base (HL), mononuclear metal complexes and standard drug. Agar well diffusion technique was used to evaluate the antibacterial activity of synthesized compounds.

|  | *Fungi* | | *Gram +* | | *Gram -* | |
| --- | --- | --- | --- | --- | --- | --- |
|  | *C. albicans* | *A. fumigatus* | *S. aureus (+ve)* | *B. subtilis (+ve)* | *E. coli  (-ve)* | *P. vulgaris  (-ve)* |
| **CuL** | 14 | 20 | 10 | 21 | 12 | 14 |
| **NiL** | 12 | 17 | 9 | 21 | 0 | 12 |
| **ZnL** | 11 | 16 | 0 | 14 | 0 | 10 |
| **VOL** | 10 | 0 | 0 | 12 | 0 | 0 |
| **HL** | 10 | 0 | 0 | 10 | 0 | 0 |
| *Gentamycin* | 0 | 0 | 24 | 26 | 30 | 25 |
| *Ketoconazole* | 20 | 17 | 0 | 0 | 0 | 0 |

**11.** **In vitro anti-inflammatory activity**

**Table S4**. Effect of test solutions on Protein denaturation assay for anti-arthritic activity

| **Concentration**  **μg/ml** | **% Of inhibition** | | | | | |
| --- | --- | --- | --- | --- | --- | --- |
|  | **CuL** | **NiL** | **ZnL** | **VOL** | **HL** | **Ibu** |
| 100 | 71 | 69 | 45 | 35 | 16 | 73 |
| 200 | 74 | 72 | 55 | 40 | 19 | 78 |
| 300 | 78 | 75 | 70 | 44 | 24 | 81 |
| 400 | 90 | 88 | 73 | 59 | 26 | 93 |
| 500 | 94 | 91 | 76 | 65 | 30 | 100 |
